# Predicting daily lactation costs of marine mammals for use in bioenergetic models

**DOI:** 10.1371/journal.pone.0352443

**Published:** 2026-07-29

**Authors:** Elizabeth A. McHuron

**Affiliations:** Cooperative Institute for Climate, Ocean, and Ecosystem Studies, University of Washington, Seattle, Washington United States of America; Alaska Pacific University, UNITED STATES OF AMERICA

## Abstract

Empirical estimates of energetic processes are limited or absent for many marine mammal species, hindering the ability to quantify disturbance-related impacts on population dynamics using bioenergetic approaches. In this study, published estimates of mass-specific milk intake rates and milk energy density from marine mammals and taxonomically related terrestrial mammals were used to examine how these variables changed as a function of the percentage of time into the lactation interval. Predictions of milk energy intake derived from these relationships were compared with independent estimates from other approaches to inform their utility in estimating daily lactation costs of data-poor species. Daily mass-specific milk intake rates declined between the start and end of lactation for all taxonomic groups, but the rate of decline was much steeper for ursids, artiodactyls, and mustelids than it was for otariids or phocids. Similarly, most taxonomic groups exhibited some increases in milk energy density as lactation progressed, but the magnitude and timing varied among families. Comparisons with independent estimates revealed the likely utility for using the derived relationships in estimating daily lactation costs of data-poor phocids, otariids, and mustelids, with some caveats. There was a large mismatch in the temporal pattern between independent estimates for cetaceans and predictions derived from semi-aquatic and terrestrial species, indicating that cetaceans have higher mass-specific milk intake rates early in lactation compared to most other species that then rapidly decline during the first 10–25% of the lactation interval. Such differences may be an adaptation to a fully aquatic lifestyle and highlight the importance of other methods for estimating daily lactation costs of data-poor cetaceans in bioenergetic models.

## Introduction

Marine mammals are exposed to a multitude of natural and human-driven stressors, many of which are increasing in magnitude and frequency [1,2]. Many of these stressors influence individuals through energetic pathways, such as the influence of marine heatwaves on prey availability or the effects of avoidance behavior or landscape changes on time-activity budgets [3–5]. Altered energy balances of individuals can influence population dynamics through changes in reproductive success and survival [6–9], as illustrated by the population consequences of disturbance (PCoD) framework [10,11]. Empirical measurements of the energetic costs of physiological processes are limited or completely lacking for many marine mammal species [12], which can make it particularly challenging to develop bioenergetic models where parameter values are based on species-specific estimates. Even for bioenergetic models that rely on generalized functional relationships (e.g., dynamic energy budget models), empirical estimates are still useful for model parameterization and tuning. Knowledge of energy requirements at different ages and life history stages can also inform proper nutrition of animals managed in human care that may play an important role in the management and conservation of wild populations [13,14].

Reproduction is perhaps the costliest life history event experienced by mammals, primarily due to energy investment in offspring in the form of milk [15,16]. Total energetic investment in offspring post-parturition relies on the interplay between the rate of milk delivery, the energy density of milk, and the duration of lactation, which may be influenced by phylogeny, maternal body size, and ecological factors [17–19]. Daily costs can vary considerably throughout lactation, which is one of the primary reasons why interspecific comparisons and allometric relationships are typically restricted to estimates of milk energy delivery at “peak” lactation [18,20]. Such relationships, while valuable, have limited usefulness for bioenergetic applications because they only represent milk energy intake at one specific time. In addition, marine mammals are often outliers in allometric relationships that include data from other taxonomic groups, which may result from the relatively extreme lactation strategies of the few species for which milk energy intake has been characterized at peak lactation [19,21]. The ability to account for temporal changes in energetic costs during lactation is often desirable because of the potential interaction between energy needs, prey availability, and the timing of stressors, particularly for species where the timing of reproduction is strongly coupled with the occurrence of seasonally abundant prey resources [22].

Lactation strategies of marine mammals are highly variable and generally well characterized for each taxonomic group, with distinct differences in lactation duration, the extent of maternal reliance on stored energy (capital) vs. active foraging (income) to support lactation costs, and the contribution of offspring to their own energy needs during the dependency period [23,24]. Empirical estimates of lactation costs from free-ranging individuals have been historically limited to a select few pinniped species where measurements of milk intake and energy density can be collected, although recent technological advancements have facilitated estimates from large baleen whales based on changes in body volume while on the breeding grounds [25]. Estimates derived from captive individuals are largely based on changes in food intake rates that does not require physical handling [26], especially for small cetaceans where there is a particular concern about disruption to the mother-calf bond. Opportunistic collection of milk samples, often from stranded, by-caught, or harvested cetaceans, has provided a broader understanding of milk energy density estimates [18,27,28], but in isolation these values provided limited information about total lactation costs.

The objective of this study was to explore changes in milk intake rates and milk energy density across lactation using existing data from terrestrial and marine mammal species, with the goal of developing predictions of daily milk energy intake for use in bioenergetic models. Select terrestrial mammals were included because marine mammals comprise a diverse group of species from three orders that share functional and ecological similarities but are not necessarily closely related [29]. Cetaceans are part of the order Artiodactyla that includes even-toed ungulates [30], with hippopotamus (*Hippopotomus amphibius*) being their closest living relatives. Sirenians comprise their own order, with Proboscidea (elephants) as their closest living relatives [31], whereas all other marine mammals are part of the order Carnivora [32]. The motivation for this study was not necessarily to develop predictions for species that have been well studied, but to explore whether relationships based on these species could provide meaningful estimates or insights about daily milk energy intake for species where empirical estimates are very limited or unlikely in the foreseeable future.

## Materials and methods

### Developing predictive relationships

Literature searches using Google Scholar were conducted to identify published values of milk intake and energy density. Search terms included one or more of the following, either alone or in conjunction with other terms (e.g., species or family names): “milk”, “milk intake”, “milk composition”, “milk energy”, “labeled water”, “tritium”, and “deuterium”. Citation mining was also used to identify any additional relevant studies missed in initial searches. Studies were limited to marine mammals and terrestrial species in the orders or families Artiodactyla, Proboscidea, Mustelidae, and Ursidae, with the exception that major dairy species (cows, sheep, goats, and camels) were excluded because they are often selectively bred to increase milk production. Data from terrestrial mammalian families (other than ursids and mustelids) were not included because they do not include any representative marine mammals, are much smaller than marine mammals, and tend to have litters with multiple offspring, which is uncommon for all marine mammals except polar bears (*Ursus maritimus*) and marine otters (*Lontra felina*). To minimize methodological variation, studies reporting on milk intake rates were only included if they used labeled water, as opposed to other approaches such as weighing offspring before and after suckling. Methodological variation was not further discriminated but was present because labeled water can be used in different ways (e.g., isotope dilution vs. transfer, single vs. doubly labeled) and with different assumptions and equations to translate measurements into estimates of milk intake rates that can impact calculated values [33].

Data on daily milk intake rates, offspring mass, milk energy density, and offspring age (or time since parturition) were compiled from each source at the finest resolution possible. For species with multiple offspring, data were compiled at the scale of the individual and not the litter (e.g., average daily milk intake rate per cub). Data were largely aggregated at the species level, with the following exceptions for species that were further separated: caribou and reindeer (both *Rangifer tarandus*), Australian (*Arctocephalus pusillus doriferus*) and Cape fur seals (*Arctocephalus pusillus pusillus*), and red deer (*Cervus elaphus*) based on geography. In most cases, data at the level of the individual were directly provided within the text or associated table or could be extracted from figures using the R package *digitize* v.0.0.4 [34]. When this was not possible, information on variation in each of the relevant parameters was also compiled. If not provided, energy density was calculated from the proximate composition of milk using conversion values of 38.1 kJ g^-1^ fat, 24.5 kJ g^-1^ protein, and 16.5 kJ g^-1^ carbohydrates [35]. In several instances where the data were not available in a useable form, the study authors were contacted to request access to the data. Other relevant information was also sourced from published literature, such as lactation duration to enable comparisons among species with disparate lactation durations. Lactation duration estimates were intended to capture the average time at which the offspring is no longer nursing and not the age at which the weaning process begins. A single value was used per species.

Compiled datasets are provided in S1 Table (milk intake) and S2 Table (milk energy density), with summaries in Table 1. These datasets should not be considered exhaustive due to a variety of reasons, including that not all references were accessible, and that data were not always provided with sufficient information or in a way that was necessary given the study objectives. For example, there are milk energy density data for a considerable number of cetaceans, but most of these studies only provided rough estimates of lactation stage and were therefore not included. Many of these estimates of cetacean milk energy density can be found in the review by Oftedal [18] as well as more recent species-specific publications [27,135,136]. To account for differences in the resolution of compiled datasets (i.e., individual values vs. means), simulated data were randomly generated from a normal distribution for data points that represented mean values. This process was repeated 50 times to model uncertainty in predictions associated with the generation of random points. Each of the 50 datasets included the same values for each data point that already represented an individual value but different randomly simulated data points.

**Table 1:** Summary of milk intake and energy density data sourced from the literature. Data include the average length of the lactation period (Lactation, days), the number of unique milk intake measurements (n), and the temporal coverage of measurements across the lactation period (Coverage). Coverage was calculated as the difference between the maximum and minimum age estimates (as a % of time into the lactation interval that ranged from 3% to 100%), rescaled to a total maximum coverage of 0.97. A value <1 indicates data were available from a single point estimate.

Taxonomic group	Lactation (days)	Intake		Energy density		References
		n	Coverage (%)	n	Coverage (%)	
*Bovidae*						
Bongo	290			25	97.8	[36]
Muskox	243	16	36.5	23	47.3	[37,38]
						
*Camelidae*						
Alpaca	183			398	82.0	[39–42]
Llama	189	53	92.2	47	99.7	[41,43–45]
*Cervidae*						
Caribou	150	12	59.3	11	58.5	[37]
Moose	183	36	64.4	56	67.8	[46]
Red deer	243			10 - 126	96.8 - 98.9	[47–49]
Reindeer	150	21	15.1	161	96.2	[50–55]
Rocky mountain elk	150	81	50.9			[56]
*Delphinidae/Phocoenidae*						
Bottlenose dolphin	304			62	91.4	[57–60]
Harbor porpoise	1,045			6	7.8	^a^
P. white-sided dolphin	549			8	13.1	[61]
*Elephantidae*						
African elephant	1,095			30	87.2	[62–64]
Asian elephant	1,460			77	76.7	[65,66]
*Giraffidae*						
Okapi	189			10	95.5	[67]
Reticulated giraffe	392			13	35.8	[68,69]
*Mustelidae*						
American mink	42	110	51.5	36	54.0	[70,71]
Domestic ferret	42			120	83.4	[72]
*Otariidae*						
Antarctic fur seal	116	249	85.1	153	99.5	[73–75]^b^
Australian fur seal	313	46	86.9	66	99.9	[76,77]
Australian sea lion	526			67	86.6	[78,79]
California sea lion	319	43	6.5	25	69.5	[80–82]
Cape fur seal	304	13	<1.0	15	<1.0	[83]^c^
Galapagos sea lion	335			1	<1.0	[84]
Juan Fernandez fur seal	274			18	36.5	[85]
New Zealand sea lion	304			257	10.2	[86]^c^
Northern fur seal	123	103	62.2	153	84.5	[87–89]^b^
Southern sea lion	274			4	<1.0	[90]
Steller sea lion	335	23	4.8	51	<1.0	[91,92]
Subantarctic fur seal	304	14	<1.0	75	83.4	[74,93,94]
*Phocidae*						
Bearded seal	24	3	6.4	4	68.7	[95]
Crabeater seal	17	3	51.5	7	91.0	[96]
Gray seal	17	90	78.8	176	90.8	[97–100]
Harbor seal	24			40	74.6	[101]
Harp seal	12	20	61.4	42	94.5	[102–104]
Hooded seal	4	11	11.6	23	77.3	[104–107]
Northern elephant seal	26	66	19.8	42	99.1	[108–113]^b^
Ringed seal	39	11	68.7	2	44.9	[114,115]
Southern elephant seal	23			145	98.6	[116–119]
Spotted seal	18			7	68.7	[120]
Weddell seal	42	39	41.7	128	99.5	[121–123]
*Ursidae*						
American black bear	475	68	56.5	85	62.4	[124–126]
Brown bear	840	49	31.1	28	30.9	[125]
Giant panda bear	639			3	21.7	[127,128]
Polar bear	913	14	40.0	116	92.8	[129–131]
Sun bear	840			1	<1.0	[132]

^a^ Unpublished data provided by Cara Gallagher (Aarhus University) and Paulien Bunskoek (Dolfinarium Harderwijk)

^b^ Individual milk intake measurements provided by J.P.Y. Arnould, D. Crocker, M. Donohue, and B. McDonald

^c^ Data acquired from theses but published in [133,134]

^d^ [118] originally published as fur seal milk

Generalized additive models (GAMs) were used to examine temporal variation in daily milk intake rate (g milk day^-1^ g offspring^-0.82^) and milk energy density (kJ g^-1^) using the “gam” function in the *mgcv* package v. 1.9−1 [137]. An exponent of 0.82 was used based on Riek [21], who found this was an appropriate scaling exponent for body mass of suckling mammals. Estimates were excluded if they occurred <3% of the way into lactation or during the perinatal period (otariids only). This was because one or both response variables is known to be different very early in lactation, this period was largely underrepresented in the compiled data, and variation could not be captured well because it was such a short temporal period for many species. Separate milk intake rate models were run for each of the following five taxonomic groupings: artiodactyls, mustelids, otariids, phocids, and ursids. Artiodactyls were grouped into a single analysis because two of the three families within this order only consisted of data from a single species. Separate milk energy density models were run for each family except dolphins and porpoises that were combined into a single taxonomic grouping. Predictor variables included in each model were a smoothed effect for the proportion of time into the lactation interval and a random effect for species (bs = “re”), which allowed for species-specific intercepts but not smooths. Model specifications include a gaussian distribution with a log (milk intake) or identity (milk energy) link function, and REML to estimate the smooth parameter. The parameter *k*, which controls how complex or “wiggly” a smooth can become, was fixed at five to minimize added complexity that largely appeared to result from unequal distribution of species or studies across the lactation period. Individual models were run for each replicate dataset when present (i.e., replicate datasets were not necessary for every taxonomic grouping). Assumptions were checked via residuals plots generated using the *gratia* package v. 0.9.2.9010 [138].

Predictions of milk intake rates and energy density were made for each species and replicate (when necessary) at 0.1% intervals using the “predict_gam” function in the *tidygam* package v. 1.0.0 [139]. These predictions and associated confidence intervals can be found in S3 Table (milk intake), S4 Table (milk energy density – non-carnivores), and S5 Table (milk energy density – carnivores). For presentation in figures, predictions at each interval were averaged across all replicates. Species-specific predictions overlayed on empirical data can be found in S1-S14 Figs.

### Assessing predictive relationships

The primary goal of this study was to understand whether the derived relationships could be useful in predicting daily milk energy intake in marine mammals that are data poor. While this requires some exploration of appropriate proxies for species without full taxonomic representation in the models, the ability to do so in any sort of rigorous or systematic fashion is hindered by the lack of independent data for comparison. Data from five marine mammal species across four families —southern elephant seals (*Mirounga leonina*), spotted seals (*Phoca largha*), sea otters (*Enhydra lutris*), bottlenose dolphins (*Tursiops truncatus*), and humpback whales (*Megaptera novaeangliae*) — that had limited (or no) representation within the analysis were used to provide a preliminary assessment of how model predictions of milk energy intake compared with estimates derived using other methods. Deviations of model predictions from independent estimates, described in further detail below, should not be universally interpreted as poor predictive performance. Rather, they are largely meant to provide a semi-quantitative assessment of how similar model estimates are to existing estimates to inform the application of the study results for estimating milk energy intake for data-poor species. Independent estimates have caveats, and while some form of empirical data underly all these estimates, they were often a more indirect estimate of milk energy intake that relied on various assumptions to generate that estimate. It was also not always possible to exactly replicate the underlying parameters the independent estimate was based on, meaning that perfect alignment was often not expected.

Comparisons were made by calculating the absolute and relative deviations between predictions and independent estimates at a daily timescale and/or summed across the measurement period. Where applicable, similarity was also assessed by calculating the estimated area of overlap between kernel density estimates of independent estimates and predictions using the *overlapping* package v. 2.1 [140]. For phocids, predicted estimates were based on combinations of milk intake rate and energy density predictions for the six phocid species represented in both analyses (e.g., northern elephant seal milk intake with northern elephant seal milk energy density), in addition to combinations of milk intake rates from each species with milk energy density predictions of the target species. For the mustelid and two cetaceans, predicted estimates were based on milk intake rate and energy density predictions for all species represented in both analyses, regardless of taxonomic representation. This was because there was no direct taxonomic representation for cetaceans in the analysis, and the representation for sea otters was from a terrestrial species. In addition, milk intake rates were combined with milk energy density estimates from the target species (bottlenose dolphins) or species in the analysis that presumably had similar milk energy density estimates as the target species (sea otter, humpback whale).

*Southern elephant seals*: The independent estimate came from two sources [141,142], which both measured maternal mass loss across lactation in this capital-breeding species. These two studies appeared to use data from the same individuals, but present output in slightly different ways. Data from a third study [143] were not included because estimates of maternal energy expenditure were not provided, which are necessary to separate maternal maintenance from milk costs. Maternal mass loss estimates, provided as either mean total energy loss (4,414 MJ) or daily mass loss rates (7.9 kg day^-1^), were subtracted from mean energy expenditure estimates (1,965 MJ or 3.4 kg day^-1^). The daily mass loss rate attributed to lactation was multiplied by the average energy density of mass loss (23.1 MJ kg^-1^; [141]) and a lactation duration of 23 days. Estimates were multiplied by 0.95 to account for any inefficiencies in milk transfer. Predictions were made based on a lactation duration of 23 days and birth mass and growth rate estimates from Arnbom et al. [142]. Mass was estimated separately for male and female pups and then averaged for each day. Predictions were made for each replicate model, averaged, and then summed across lactation to produce a single estimate of milk energy intake that could be compared with the independent estimate.

*Spotted seals*: The independent estimate came from a single spotted seal pup that was fed milk collected from a lactating female in the same facility [120]. The total amount of milk delivered daily as well as the daily energy density of milk was measured, which was also the sole source of data for this species in the milk energy density analysis. While this does not represent ‘natural’ nursing, the authors state that attempts were made to mimic the natural frequency of nursing in spotted seals and the mass gain of this pup was not statistically different from six other pups housed in the same facility that were nursing from their mothers. Predictions were based on a lactation duration of 18 days derived from the six naturally-nursed pups, but total costs were truncated to the first ten days because mass was not monitored from day 11–13 when the pup received a combination of milk and solid food. Predictions were calculated similarly as described above for southern elephant seals except that comparisons with the independent estimates were made for both daily and total lactation costs. Daily costs were also used to estimate the degree of overlap between predictions and the independent estimate.

*Sea otters*: The independent estimate was based on a bioenergetic model that was parameterized using age- and behavior-specific metabolic rates from respirometry of sea otters managed in human care and activity budgets of wild sea otters [144]. Daily estimates, provided by the study author, were multiplied by 1.19 to account for additional costs of growth, digestion, and waste production. This value was derived from Cortez et al. [145], who estimated total average daily energy intake of sea otter pups < 4 weeks of age by adding these additional costs to those of Thometz et al. [144]. Predictions were based on a lactation duration of six months and the same daily mass estimates of Thometz et al. [144]. Species with maximum milk energy density estimates from 11–13 kJ g^-1^ were used for comparisons, based on milk energy density estimates from seven sea otters in various stages of lactation [146]. Comparisons and overlap estimates were truncated to the first 28 days of lactation because energy intake of the pup is solely from milk during this time [147].

*Bottlenose dolphins*: The independent estimate came from three sources, all based on bottlenose dolphins managed in human care with food intake estimates across the entire lactation period [26,148,149]. Reddy et al. [148] provided monthly estimates of mass-specific daily energy consumption for three lactating dolphins, which were digitized and converted to absolute estimates using the provided body mass for each dolphin. The average pre-pregnancy energy consumption by each dolphin was subtracted from the total estimate to isolate likely costs associated with milk intake by offspring. Data from four individuals and a total of 8 reproductive events were provided across the other two papers, presented as total monthly food consumption (in kg). Energy consumption was calculated similarly, with the exception that monthly values were scaled to a daily rate and then converted to energy based on descriptions of diet composition and prey energy density provided in the text. All estimates were multiplied by 0.95 to account for any inefficiencies in milk transfer. There were several instances where estimated costs were negative (total energy consumption during lactation was < pre-pregnancy consumption); these were excluded from the analysis since this it was assumed these were likely not representative of what would occur in wild individuals. Predictions were made based on a lactation duration of 2.2 years, which was the estimated average weaning age of captive dolphins in the three studies. Length at age was estimated based on an equation from Neuenehoff et al. [150] and a length-to-mass conversion from Mallette et al. [151]. No data on calf mass during nursing were provided, however, the resulting mass estimates produced by these equations resulted in similar mass estimates as 2-year-olds from Kastelein et al. [26]. Overlap was assessed for each reproductive event.

*Humpback whales*: The independent estimate was based on a bioenergetic model that relied primarily on data collected from mother-calf humpback whale pairs via photogrammetry and other information collected from UAS to parameterize metabolic expenditure and somatic growth across the first 90 days of lactation [25]. Daily estimates of milk energy transfer (MJ day^-1^), provided by the study author, were compared with model predictions based on calf mass estimates (also provided by study author) and a lactation duration of 10 months. Predictions were restricted to the duration of the independent estimate. As the maximum milk energy density measured in this species is ca. 20 kJ g^-1^ based on fat and protein composition [18], species with maximum milk energy density estimates between 19 and 21 kJ g^-1^ were used for comparisons.

## Results

### Milk intake rate

**Fig 1:**
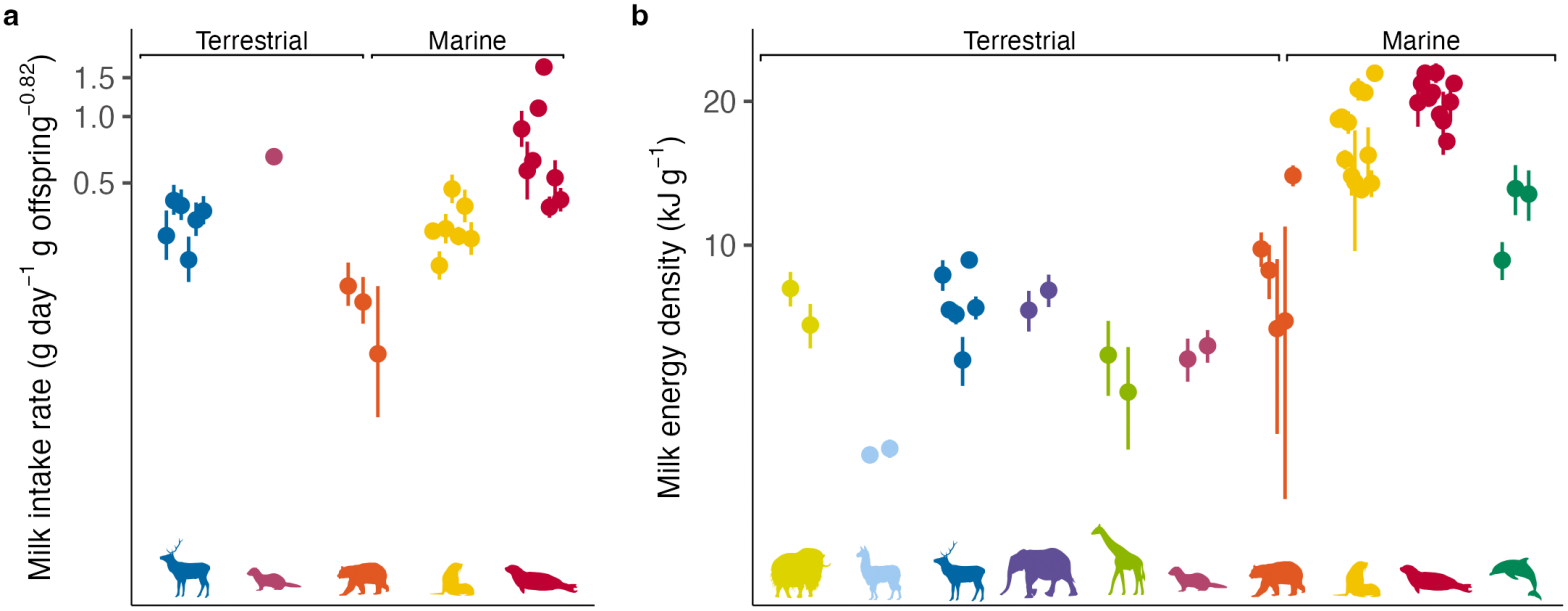
Species-specific predictions of mass-specific daily milk intake rate (a) and milk energy density (b). Values are averaged by species across the lactation interval (3–100%) and replicate populations. Error bars represent the mean 95% confidence interval. Taxonomic groupings are as follows (left to right): (a) Artiodactyla, Mustelidae, Ursidae, Otariidae, and Phocidae, (b) Bovidae, Camelidae, Cervidae, Elephantidae, Giraffidae, Mustelidae, Ursidae, Otariidae, Phocidae, and Delphinidae/Phocoenidae. Organism silhouettes are from PhyloPic (https://www.phylopic.org/; T. Michael Keesey, 2023) and were added using the rphylopic R package v.1.5.0 [152]. Silhouettes were made and contributed by the following people under CC0 1.0: Laura Barbero-Palacios (2023), Unknown (2024, contributed by Mozillian), Steven Traver (2012), An Ignorant Atheist (2019, contributed by Jody Taylor), Ferran Sayol (2015), Tracy A. Heath (2013), Alexandre Vong (2018), and Unknown (2019, contributed by Michael Day).

**Fig 2:**
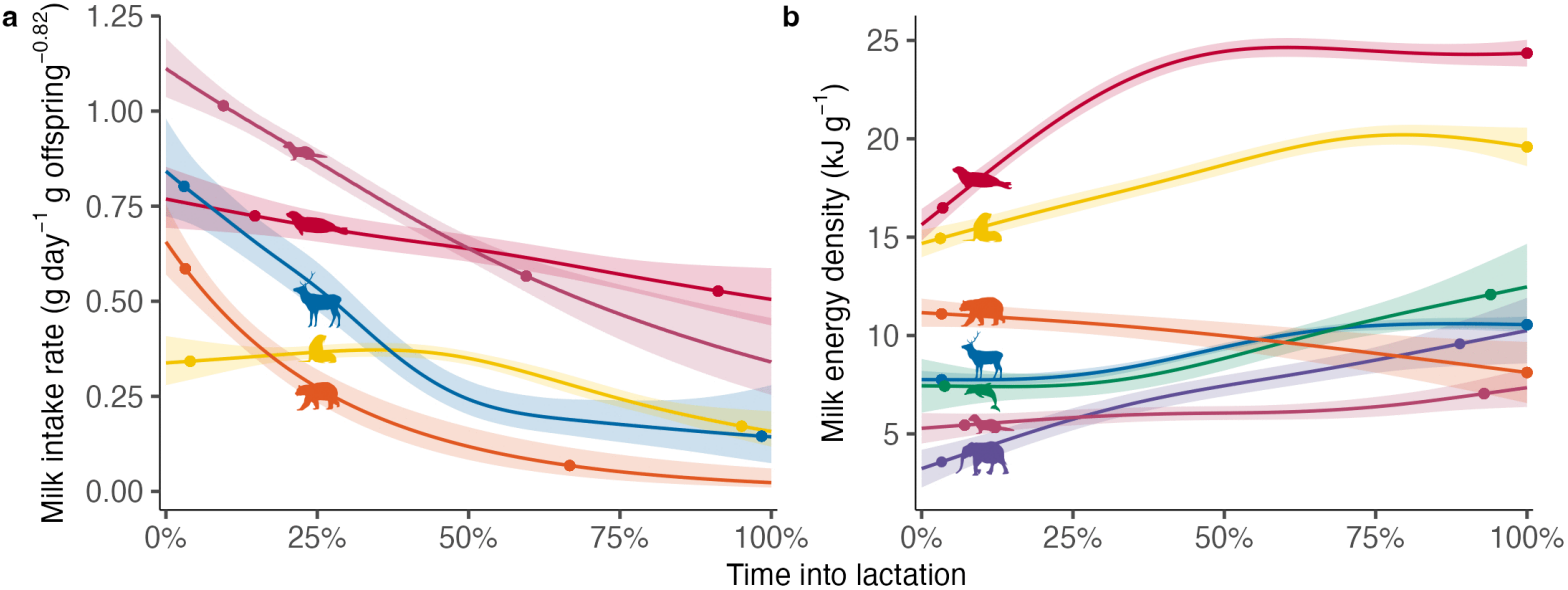
Species-specific predictions of mass-specific milk intake rates (a) and milk energy density (b) as a function of the relative time into lactation (% of the total duration). Lines represent mean predictions and shaded areas the mean 95% confidence interval, averaged across all replicate models. Dots correspond to the minimum and maximum times included in the analysis for each family; predictions outside of these dots correspond to extrapolation beyond the model limits. In b, not all taxonomic groups are shown for ease of visualization. Predictions are based on the following species (top to bottom): (a) American mink (Mustelidae), gray seal (Phocidae), reindeer (Artiodactyla), Antarctic fur seal (Otariidae), American black bear (Ursidae); (b) gray seal (Phocidae), Antarctic fur seal (Otariidae), American black bear (Ursidae), reindeer (Cervidae), bottlenose dolphin (Delphinidae/Phocoenidae), domestic ferret (Mustelidae), and African elephant (Elephantidae). Organism silhouettes are from PhyloPic (https://www.phylopic.org/; T. Michael Keesey, 2023) and were added using the rphylopic R package v.1.5.0 [152]. Silhouettes were made and contributed by the following people under CC0 1.0: Ferran Sayol (2015), Alexandre Vong (2018), Tracy A. Heath (2013), Steven Traver (2012), and Unknown (2019, contributed by Michael Day).

The milk intake rate dataset comprised 25 species (or subspecies) from 2 orders and 7 families (Table 1). Average species-specific sample sizes within each family were 16 (Bovidae), 53 (Camelidae), 37 (Cervidae), 110 (Mustelidae), 70 (Otariidae), 30 (Phocidae), and 43 (Ursidae). All model groupings (by family or Order in the case of Artiodactyla) exhibited inter-specific differences in mass-specific daily milk intake rates (Fig 1a, Table 2), except Mustelidae that was only comprised of a single species. There were significant temporal changes in mass-specific milk intake rates for all family or order groupings (Fig 2a), and while all models exhibited declines between the start and end of lactation, the specific temporal patterns and magnitude of the decline differed considerably among species groups. Artiodactyls, mustelids, and ursids exhibited considerable declines in mass-specific milk intake rates across the first half of lactation, which contrasted with patterns exhibited by phocids and otariids where temporal changes across lactation were of a smaller magnitude (Fig 2a). When averaged across lactation, mean daily mass-specific milk intake rates were generally highest for phocids and mustelids, intermediate and similar between otariids and artiodactyls, and lowest for ursids (Fig 1a).

**Table 2:** Summary of generalized additive models for milk intake rate and milk energy density analyses. The number of observations, the percent deviance explained by the model, and the individual percent contribution of each model term to the explained deviance [153] are shown. Model terms were a random effect for species (or subspecies) and a smoothed effect for the relative time into lactation. For taxonomic groups where multiple models were run using data simulated from mean values, the standard deviation (deviance) or range (species, time) among models is shown.

Model	n_Obs_	Deviance (%)	Species (%)	Time (%)
*Intake*				
Artiodactyla	165	71.6 ± 4.9	13.7 (12.1–16.9)	86.3 (83.1–88.0)
Mustelidae	110	66.3	–	100.0
Otariidae	491	38.5 ± 1.5	48.7 (45.5 – 53.7)	51.3 (46.3–54.5)
Phocidae	243	82.5 ± 1.0	96.7 (93.1–98.5)	3.3 (1.5–6.9)
Ursidae	131	51.3 ± 7.8	21.6 (14.6–31.1)	78.4 (68.9–85.4)
				
*Energy density*				
Bovidae	48	52.8	45.5	54.5
Camelidae	445	3.5 ± 1.1	23.0 (0–77.6)	77.0 (22.4–100)
Cervidae	396	72.1 ± 1.4	48.5 (45.4–52.0)	51.5 (48.0–54.6)
Delphinidae/Phocoenidae	76	51.1	52.9	47.1
Elephantidae	104	58.2	2.9	97.1
Giraffidae	23	18.2	85.0	15.1
Mustelidae	156	10.0 ± 5.2	29.3 (0–69.0)	70.7 (31.0–100)
Otariidae	891	81.1± 0.5	63.4 (62.6–64.6)	36.6 (35.8–37.4)
Phocidae	621	71.6 ± 1.1	47.7 (42.8–51.6)	52.3 (48.4–57.2)
Ursidae	233	46.7 ± 3.4	95.4 (92.6–97.6)	4.6 (2.4–7.6)

### Milk energy density

The milk energy density dataset comprised 47 species (or subspecies) from 3 orders and 10 families (Table 1). Average species-specific sample sizes within each family were 24 (Bovidae), 223 (Camelidae,), 66 (Cervidae), 25 (Delphinidae/Phocoenidae), 54 (Elephantidae), 12 (Giraffidae), 78 (Mustelidae), 74 (Otariidae), 56 (Phocidae), and 47 (Ursidae). Most families exhibited inter-specific differences in milk energy density, with the exception being camelids and giraffids (*p >* 0.05; Fig 1b, Table 2). Giraffids were the only family for which there were no apparent changes in milk energy density across lactation, and while significant, the magnitude of temporal changes in milk energy density of camelids was very small (≤ 1 kJ g^-1^). Almost all other families experienced increases in milk energy density across part of lactation, except for ursids that experienced a slight decline (Fig 2b). Family-specific differences were apparent in both the magnitude of the temporal changes in milk energy density as well as the timing.

### Predictions

The mean independent estimate of total milk intake for southern elephant seals was 2,299 MJ. Predicted total milk energy intake costs based on ringed seals *Pusa hispida* were closest to this estimate (2,351 MJ, + 2.3%) followed by Weddell seals *Leptonychotes weddellii* (2,241 MJ, −2.5%), and northern elephant seals *M. angustirostris* (1,784 MJ, −22.4%). The five remaining species produced estimates that were 32% – 310% greater than the independent estimate, with harp *Pagophilus groenlandicus* and hooded seal *Cystophora cristata* predictions resulting in the most disparate values. Based on combinations with southern elephant seal milk energy density predictions, daily average mass-specific milk intake rates for this species appear to fall between 0.57 and 0.64 g day^-1^ g offspring ^−0.82^.

**Fig 3:**
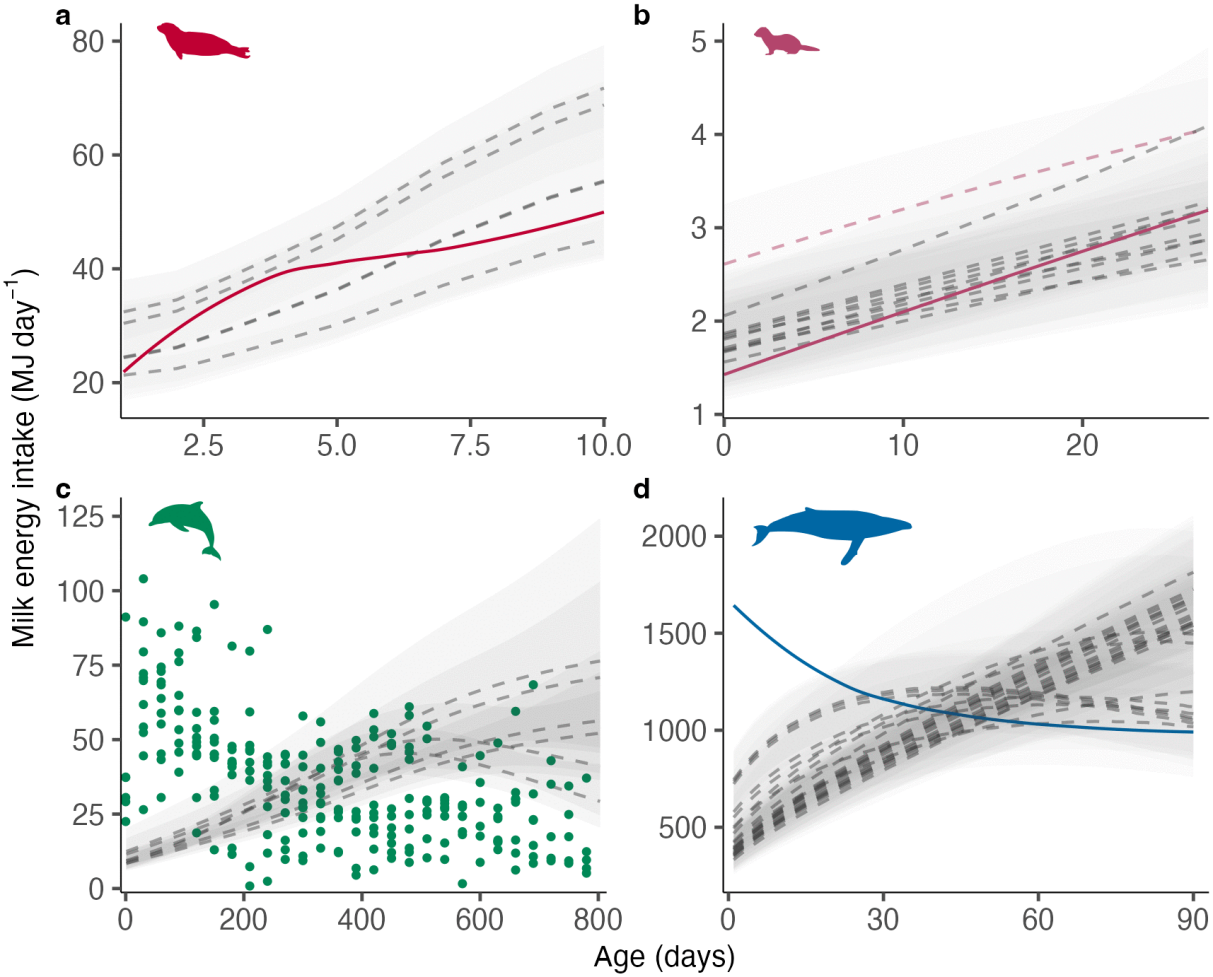
Comparisons of predicted daily milk energy intake (dashed lines) with independent estimates (solid lines or points) derived from the literature for spotted seals (a), sea otters (b), bottlenose dolphins (c), and humpback whales (d). Each prediction represents a single species-specific combination of milk intake rates with milk energy density predictions from the same species, or a species with milk energy density of a similar magnitude as the target species. In a and c, only predictions with overlap > 0.6 are shown, while in b predictions based on the only mustelid in the analysis is also shown despite low overlap (dashed pink line). In d, predictions with overlap between 0.5 and 0.6 are shown. Shaded areas represent 95% confidence intervals, calculated by combining lower and upper 95% CI from point estimates of milk intake rates and milk energy density estimates. Organism silhouettes are from PhyloPic (https://www.phylopic.org/; T. Michael Keesey, 2023) and were added using the rphylopic R package v.1.5.0 [152]. Silhouettes were made and contributed by the following people: Tracy Heath (2013, CC0 1.0), Ferran Sayol (2015, CC0 1.0), Unknown (2019, contributed by Michael Day, CC0 1.0), and Chris Huh (2011, contributed by T. Michael Keesey, CC BY-SA 3.0).

Spotted seal milk energy intake from the independent estimate totaled 395.2 MJ across the first ten days of lactation, with daily rates of 2.2–5.0 MJ day^-1^. Predictions of total milk energy intake based on bearded seals *Erignathus barbatus* were closest to this estimate (391.2 MJ, −1.0%) followed by gray seals *Halichoerus grypus* (325.5 MJ, −17.6%) and harp seals (511.0 MJ, + 29.3%). The combination of bearded seal milk intake rates and spotted seal milk energy density produced a nearly identical estimate to the one using predictions solely from bearded seals (392.6 MJ, −0.7%), whereas hooded seal predictions resulted in total costs that were the most different. Based on combinations with spotted seal milk energy density predictions, daily average mass-specific milk intake rates for this species appear to fall between 0.88 and 1.10 g day^-1^ g offspring^-0.82^. Absolute daily differences in estimated milk energy intake based on the bearded seal predictions ranged from 1.3 MJ to 6.7 MJ (3.1% – 18.7% of actual daily milk intake), and overlap was > 0.6 when using estimates derived from bearded, harp, and gray seals (Fig 3a).

The average and total milk intake of sea otter pups across the first 28 days of lactation from the independent estimate was 2.3 MJ day^-1^ and 65.0 MJ, respectively. Predictions derived from a terrestrial mustelid were on average 48.8% (3.4 MJ, daily) and 45.2% (94.4 MJ, total) greater than these estimates. In contrast, predictions using milk intake rates from species in other families, predominately otariids, generally produced daily estimates that were within 10% of the independent estimate (Fig 3d). Almost all of these were based on combinations with milk energy density estimates derived from other species that presumably had similar values as sea otters. Of the pure species combinations, predictions from Australian fur seals provided the closest estimates, with values that were within an average of 10.6% (daily, range of <1% –17.6%) or 4.5% (total) across the first 28 days. The overlap analysis revealed similar patterns, with predictions derived from Australian fur seals exhibiting overlap > 0.9, followed predominately by other otariids when paired with species with maximum milk energy densities between 11 and 13 kJ g^-1^. Despite low overlap, the temporal pattern of changes in daily milk energy intake was very similar between the independent estimate and predictions derived from terrestrial mustelids (Fig 3b).

The average milk energy intake across all eleven dolphin reproductive events, as estimated from food consumption, was 34.3 MJ day^-1^, with a mean maximum estimate across the three studies of 82.6 MJ day^-1^. There were 6 (of 1,104) combinations that result in overlap > 0.6 for at least 7 reproductive events. All these combinations resulted from milk intake rates estimated derived from pinnipeds, with five resulting from combinations with bottlenose dolphin milk energy density predictions. The average daily milk intake rate from these five predictions was 0.48 g day^-1^ g offspring^-0.82^ (range of 0.39 to 0.57). Overlap did not necessarily result from similar temporal changes throughout lactation, as predictions based on pinnipeds estimated higher milk energy intake later in lactation, largely contrary to estimates based on food consumption (Fig 3c). Regardless of the magnitude of milk energy intake and overlap, there was no combination that produced a similar trend as derived from the independent estimate.

**Fig 4:**
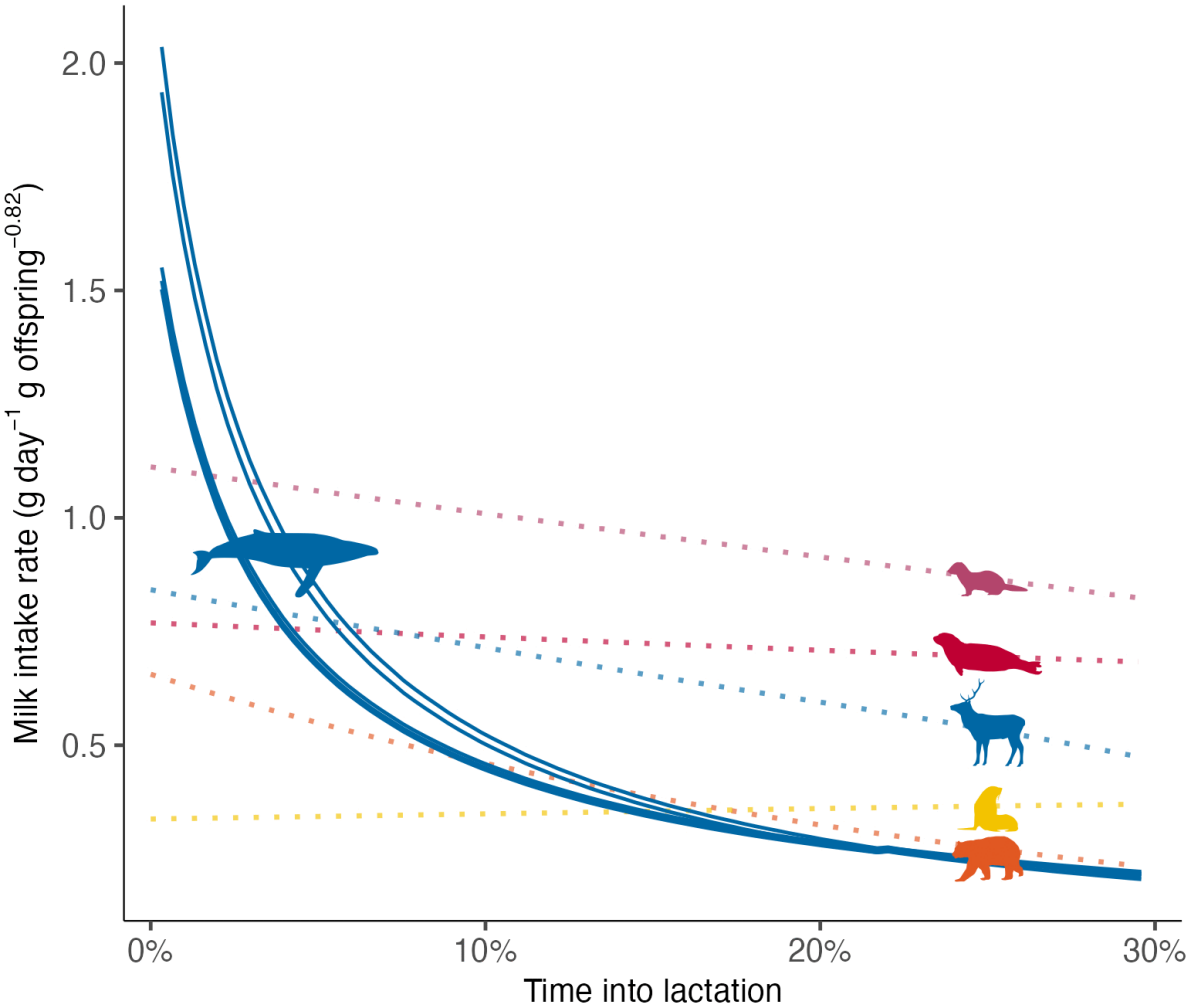
Estimates of humpback whale mass-specific milk intake rates as a function of the relative time into lactation (% of the total duration). Estimates were derived from calf milk energy transfer and mass from Christiansen et al. [25] and predicted milk energy density estimates from five species with similar milk energy density values as humpback whales. Predictions from the American mink (Mustelidae), gray seal (Phocidae), reindeer (Artiodactyla), Antarctic fur seal (Otariidae), and American black bear (Ursidae) are also shown. Organism silhouettes are from PhyloPic (https://www.phylopic.org/; T. Michael Keesey, 2023) and were added using the rphylopic R package v.1.5.0 [152]. Silhouettes were made and contributed by the following people: Ferran Sayol (2015, CC0 1.0), Alexandre Vong (2018, CC0 1.0), Tracy A. Heath (2013, CC0 1.0), Steven Traver (2012, CC0 1.0), and Chris Huh (2011, contributed by T. Michael Keesey, CC BY-SA 3.0).

Milk energy intake of humpback whales from the independent estimate totaled 103,252.7 MJ across the first 90 days of lactation, with daily estimates of 984.0 MJ – 1,725.6 MJ. On average, milk intake rates derived from ursids produced the most similar daily values, however, none of these were within 10% and all but one of the overlap estimates were < 0.6 (Fig 3d). As with bottlenose dolphins, temporal patterns in milk energy intake were not well aligned; all predictions resulted in increases in daily milk energy intake across the first part of lactation, whereas the independent estimate indicated a decline during early lactation despite mass gains by the calf across this period (Fig 3d). When considering likely milk energy density estimates of humpback whales, milk intake rate data from hooded and harp seals provided the most similar estimates at the beginning of lactation. Back calculations using the independent estimate, calf mass estimates, and milk energy density curves derived from species with similar values as humpback whales (maximum values of 19–21 kJ g^-1^) resulted in mass-specific milk intake rates of 1.52–2.04 g day^-1^ g offspring^-0.82^ at the start of lactation that rapidly declined to 0.21 or 0.22 g day^-1^ g offspring^-0.82^ at 90 days, with an overall average of 0.45–0.54 g day^-1^ g offspring^-0.82^ (Fig 4).

## Discussion

Interspecific variation in mammalian lactation costs has been well documented, particularly as it relates to the high rates of milk energy transfer by phocid seals and the generally higher milk fat content of marine compared with terrestrial mammals [18,19,154]. While the purpose of this paper was not to provide a rigorous analysis of interspecific variation in milk intake rates and energy density, the resulting predictions indicate that many of these patterns hold when assessed across the lactation interval and not just at peak lactation. Temporal patterns contributed to these interspecific differences, as phocids (and otariids) exhibited much shallower declines in mass-specific milk intake rates compared with all other taxonomic groups. While average milk intake rates of otariid seals were similar to those of terrestrial artiodactyls, this comparison is somewhat misleading because many otariid pups do not nurse every day. Per nursing day, mass-specific milk intake rates for many otariids included in the analysis are likely to be 2–3 times greater than predictions based on the measurement interval [75]. In this context, otariid milk intake rates are more in-line with phocids despite family-specific differences in the reliance on energy obtained during (income) vs. stored prior to (capital) lactation to support milk production. Taxonomic differences in overall averages and temporal patterns are likely to be driven by a variety of factors, including maternal behavior (e.g., fasting vs not fasting), offspring growth strategies, physical constraints (e.g., stomach size), and how much (if any) offspring supplement their diet with solid food [17,19,23,154,155].

The potential utility for predicting lactation costs of data-poor marine mammal species using the derived relationships appears variable based on comparisons with independent estimates. Taxonomic-specific applications, discussed in more depth in the following section, are largely intended to focus on species only represented in one or neither of the analyses. Some of the marine mammal species included in both analyses would still be considered data poor given the relatively few data points available and/or limited temporal coverage across lactation, such as crabeater seals (*Lobodon carcinophaga*) and several sea lion species. Applications for these species are more straightforward since predictions still represent the best available estimates given species-specific data. For all applications, uncertainty can be incorporated through various mechanisms, such as through uncertainty in the estimates themselves or by using predictions for multiple species. As the analysis presented here is an imperfect solution to a difficult problem, additional caveats and limitations of this approach are also discussed.

### Application to data-poor species

Analyses of milk intake rates and energy density were separated here for practical reasons, both to increase the number of species that could be included in each analysis and because of temporal mismatches that result from the fact that a given study may measure one variable but not the other. While this theoretically allows for “mixing and matching” in estimating daily milk energy intake, as was done somewhat in comparisons with independent estimates, caution should be used in doing so because these two variables are highly likely to be interdependent. Otariids illustrate this issue nicely, where mean milk energy densities tended to be negatively correlated with mean milk intake rates (*r = −*0.7). Because of intermittent nursing visits, increases in milk energy density may represent a way that females can increase energy delivery to their pups to sustain them for a prolonged fast, as there is only so much milk a pup can physically consume in a 1–2-day nursing visit [156,157].

Predictive relationships derived in this study are likely to be useful in estimating daily and total milk energy intake of data-poor phocids. Spotted seal milk energy intakes were best predicted by species that bred in the same habitat type (pack ice, bearded and harp seal) and/or had very similar lactation durations (gray seals). Actual milk intake rates for spotted seals were predicted to fall between those of bearded and harp seals, which is consistent with their intermediate lactation duration between these two species. Southern elephant seal milk energy intakes were best predicted by ringed and Weddell seals, which also breed on a stable substrate (fast ice), but have comparatively longer lactation durations and use a mixed capital-income strategy during lactation [122,158]. Although these results were slightly unexpected—it was assumed that the values derived from northern elephant seals would provide the closest estimate—most species breeding in stable habitats provided more similar estimates than those breeding in unstable pack ice environments. These comparisons indicate that milk intake rates of data-poor phocids are likely best predicted using relationships from species with similar breeding substrates (land or fast ice vs pack ice) and/or lactation durations, a recommendation that is consistent with previous research highlighting the influence of breeding substrate stability on phocid lactation strategies [17,155,158]. Similarity in lactation strategy did not appear to be a necessity for selecting a proxy species, despite the influence of maternal body mass on milk energy investment in offspring of capital-breeding phocids [109].

Although an independent estimate for comparison was lacking, there is likely utility for using predictions from this study to estimate milk energy intake of data-poor otariids given the extensive data available for a few species. The use of predictive relationships to estimate otariid lactation costs does require additional consideration because the values used for each species in the milk intake rate analysis include varying levels of absence by the mother from the pup. While it would be ideal to also provide an analysis of milk intake rates per nursing day, most otariid studies do not report milk intake rates this way and may not have the data needed to do so because it requires additional monitoring during the measurement interval. Depending on the specific bioenergetic application, it may be useful to adjust mass-specific milk intake rate predictions to reflect delivery per nursing day, such as by using a simple species-specific multiplier based on the estimated nursing durations of animals included in the analysis.

There are only two extant marine mammals in the mustelid family, the sea otter and the marine otter. Because daily lactation costs of sea otters have already been well described [144,159], predictions provided here are largely useful for their application to marine otters that are data poor, endangered, and threatened by human activities [160]. Estimates of daily milk energy intake based on terrestrial mustelids were consistently higher than those of Thometz et al. [144], although the temporal pattern was similar. Instead, milk intake rates of otariids provided the closest estimates, but only when they were combined with milk energy density estimates from species with maximum values between 11 and 13 kJ g^-1^. While terrestrial mustelids are more closely related to sea otters than otariids, sea otters give birth to a single pup, which may result in lower mass-specific milk intake rates since daily milk production is influenced by litter size [161] and increased suckling stimulus associated with multiple offspring may maximize milk yield [71]. Because sea otter pups nurse daily and do not undergo intermittent fasting, it is not particularly surprising that estimates were most similar when milk intake rates from otariids were combined with milk energy densities more characteristic of sea otters than otariids. These comparisons indicate that daily milk intake rates of marine otters, which have similar lactation durations as sea otters but litters of two to four pups, may fall between the two species and be cautiously estimated using relationships derived from some otariids or by multiplying the values generated using terrestrial mustelids by a factor < 1. One additional caveat is that the data used to estimate mustelid milk intake rates did not include the latter part of lactation when offspring supplement their diets with solid food, whereas otariids do not receive the same level of supplementation (if any) in their diets as marine mustelids. This could result in overestimates of milk energy intake by offspring in the second part of lactation and predictions may require some adjustment to account for this.

Mass-specific milk intake rates derived from other terrestrial and marine mammal taxonomic groups appear unlikely to provide the ability to inform daily lactation costs of cetaceans. Comparisons do, however, provide insight into temporal patterns of mass-specific milk intake rates for cetaceans. Specifically, they indicate that cetaceans appear to have higher mass-specific milk intake rates early in lactation (< 10% − 25% of the total interval) than most other taxonomic groups, which then decline considerably as lactation progresses. This results in daily milk energy costs that are generally highest at the beginning of lactation despite continuous offspring growth, a pattern that differs from semi-aquatic and terrestrial mammals. While this assumes that the independent estimate largely represents the truth, there is other empirical support for this hypothesis, including changes in appearance, rapid growth, and intense nursing during early lactation [162–167]. For example, Christiansen et al. [164] found that southern right whale (*Eubalaena australis*) calves were slender at birth but exhibited rapid growth in body width during the first 30 days of lactation. Similarly, Ransome et al. [168] noted that newborn humpback whale calves had a thin blubber layer with visible bone structure. Blubber plays an important role in thermoregulation, locomotion, and energy storage, and there is evidence of ontogenetic changes in structure, composition, and thickness in cetaceans [169–171]. Blubber lipid content is particularly low (<10%) in very young baleen whale calves [172,173], which increases the thermal conductivity of blubber [174] and may require rapid changes to reduce thermoregulatory costs. Bottlenose dolphins are similarly thin at birth [162,163], although Cockcroft [162] found that rapid gains in the first 30 days were largely attributed to musculature gains that coincided with the development of swimming behavior. High mass-specific milk intake rates very early in lactation may thus be necessary to accommodate the unique challenges associated with a fully aquatic lifestyle while also balancing tradeoffs associated with *in-utero* development and positioning [175] and the physical constraints of birth [176].

### Limitations

Aside from uncertainty and errors associated with the methods used to estimate milk intake and energy density in the original papers, errors could have been introduced because data were not always presented in the exact way they were used in the analysis. For example, many of the data sources required digitization of data from figures that either needed to be combined (e.g., milk intake rates with mass estimates, proximate milk composition) and/or extrapolated from means to individual values. Replicate analyses were intended to incorporate some of the potential error associated with extrapolating means to individual values but cannot eliminate the issue entirely. In addition, data were combined across species regardless of the specific methodology of each study. Because analyses were conducted by pooling multiple species within a taxonomic group and the complexity of smooths was limited, these types of potential errors are most likely to be influential on predictions for species with relatively few data points through their influence on the y-intercept.

A single lactation duration was typically used for each species, but this does not reflect intraspecific variability nor was it always clear what that value should be, particularly for ursids and odontocetes where lactation durations can vary on the order of years [177,178]. In addition, many of the data from terrestrial species came from animals managed in human care where they may be purposefully weaned earlier than their free-ranging counterparts. Uncertainty in lactation durations is most likely to impact the specific timing of changes in milk intake rates or energy density during lactation but should have lesser impacts on the broader conclusions regarding taxonomic differences.

To maximize species representations and sample sizes, species were included even if only represented by a few samples and studies using a variety of methods were included in the analysis, within the initial constraints detailed in the methods. Small sample sizes may result in incomplete representation of species parameters, and pooling of data using different methodologies could result in inter- or intraspecific patterns where none truly exist due to method-specific biases [156]. This contributed to the decision to not include species-specific smooths within each analysis, which could result in species-specific inaccuracies because data-rich species contributed more to the shape of the curve. For example, the otariid milk intake analysis was heavily biased towards fur seals over sea lions, particularly two small, high latitude species with much shorter lactation durations than other otariids (ca. 4 months vs 8 months – 1 + years). Some species-specific variation is likely because the relative timing of life history events for dependent offspring (e.g., molt) and other factors that are likely to influence milk intake (e.g., supplemental feeding) varies among species within a given taxonomic group. This may result in some inaccuracies in predictions of milk energy intake at specific times that are likely of lesser magnitude than inter-specific differences. Such inaccuracies are less likely to influence conclusions regarding taxonomic differences or the inability to reproduce the temporal patterns of milk energy intake in the independent cetacean estimates. Because of limitations associated with the analysis, application to species where temporal sampling and sample sizes are robust is not intended because species-specific analyses are likely to better capture the nuances of temporal fluctuations.

Despite pooling data within taxonomic groups, some taxonomic groups still lacked representation across a significant portion of the lactation interval, particularly for milk intake rates (e.g., mustelids). Insufficient data were available to represent Sirenians [179,180] and as fully aquatic and herbivorous marine mammals, it is difficult to provide any recommendations for manatees (*Trichechus* spp.) or dugongs (*Dugong dugon*). In addition, there is often considerable variation in body size, lactation strategies, and habitat use within a given marine mammal taxonomic group, all of which may impact metabolic demands [181,182]. For example, female phocids range in body size from roughly 50 kg to 700 kg, occupy a spectrum of partial to complete dependence on a capital-breeding strategy, and occur in polar, temperate, and tropical ecosystems. This variation complicates the selection of proxy species when a species unique features are not well represented, such as for monk seals (*Monachus monachus* and *Neomonachus schauinslandi*) that inhabit warmer environments and/or exhibit considerable differences in lactation duration compared with other phocids [183]. Additional data collection on species present in the analysis or new species would thus be useful in strengthening the confidence around species predictions and informing extrapolation to data-poor species.

### Conclusion

As the most energetically expensive life history event faced by female mammals, the ability to quantify lactation costs is critical for studies investigating the effects of natural and human-induced disturbances on marine mammals. Data on milk intake rates and energy density data are valuable sources to inform these costs and as illustrated here, can help inform estimates of daily lactation costs in bioenergetic models for data-poor phocids, otariids, and mustelids. Predictions derived from these data do not appear to be a viable approach for estimating milk energy intake of cetaceans, nor should they be applied to Sirenians without some assessment of predictive performance. For cetaceans, the mismatch in temporal patterns of milk energy intake between predictions and the independent estimate indicates they have considerably higher mass-specific milk intake rates early in lactation than most semi-aquatic or terrestrial species, which may be an adaptation to a fully aquatic lifestyle. Daily lactation costs for data-poor cetaceans are thus likely to be better estimated using existing approaches or potentially by generalizing relationships derived from drone-based studies of well-studied species (mysticetes) or high-resolution data collected from animals managed in human care (odontocetes). While the focus of this paper was on marine mammals, disturbance is a universal challenge faced by all mammals and a similar approach could be used for estimating lactation costs of understudied terrestrial carnivores.

## Supporting information

S1 Fig Mass-specific daily milk intake predictions (line) and input data (circles) as a function of the relative time into lactation (% of the total duration) for species in the family Phocidae. Measurements that occurred <3% of the time into the lactation interval were not included. Subplots correspond to each species included in the analysis. The size of individual data points corresponds to the number of individual measurements associated with it, with the smallest size corresponding to a single measurement per point.
(TIFF)

S2 Fig Mass-specific milk intake predictions (line) and input data (circles) as a function of the relative time into lactation (% of the total duration) for species in the family Otariidae. Measurements that occurred <3% of the time into the lactation interval were not included. Subplots correspond to each species included in the analysis. The size of individual data points corresponds to the number of individual measurements associated with it, with the smallest size corresponding to a single measurement per point.
(TIFF)

S3 Fig Mass-specific milk intake predictions (line) and input data (circles) as a function of the relative time into lactation (% of the total duration) for species in the family Mustelidae. Measurements that occurred <3% of the time into the lactation interval were not included. The size of individual data points corresponds to the number of individual measurements associated with it, with the smallest size corresponding to a single measurement per point.
(TIFF)

S4 Fig Mass-specific milk intake predictions (line) and input data (circles) as a function of the relative time into lactation (% of the total duration) for species in the family Ursidae. Measurements that occurred <3% of the time into the lactation interval were not included. Subplots correspond to each species included in the analysis. The size of individual data points corresponds to the number of individual measurements associated with it, with the smallest size corresponding to a single measurement per point.
(TIFF)

S5 Fig Mass-specific milk intake predictions (line) and input data (circles) as a function of the relative time into lactation (% of the total duration) for species in the order Artiodactyla. Measurements that occurred <3% of the time into the lactation interval were not included. Subplots correspond to each species included in the analysis. The size of individual data points corresponds to the number of individual measurements associated with it, with the smallest size corresponding to a single measurement per point.
(TIFF)

S6 Fig Milk energy density predictions (line) and input data (circles) as a function of the relative time into lactation (% of the total duration) for species in the family Phocidae. Measurements that occurred <3% of the time into the lactation interval were not included. Subplots correspond to each species included in the analysis. The size of individual data points corresponds to the number of individual measurements associated with it, with the smallest size corresponding to a single measurement per point.
(TIFF)

S7 Fig Milk energy density predictions (line) and input data (circles) as a function of the relative time into lactation (% of the total duration) for species in the family Otariidae. Measurements that occurred <3% of the time into the lactation interval were not included. Subplots correspond to each species included in the analysis. The size of individual data points corresponds to the number of individual measurements associated with it, with the smallest size corresponding to a single measurement per point.
(TIFF)

S8 Fig Milk energy density predictions (line) and input data (circles) as a function of the relative time into lactation (% of the total duration) for species in the family Mustelidae. Measurements that occurred <3% of the time into the lactation interval were not included. Subplots correspond to each species included in the analysis. The size of individual data points corresponds to the number of individual measurements associated with it, with the smallest size corresponding to a single measurement per point.
(TIFF)

S9 Fig Milk energy density predictions (line) and input data (circles) as a function of the relative time into lactation (% of the total duration) for species in the family Ursidae. Measurements that occurred <3% of the time into the lactation interval were not included. Subplots correspond to each species included in the analysis. The size of individual data points corresponds to the number of individual measurements associated with it, with the smallest size corresponding to a single measurement per point.
(TIFF)

S10 Fig Milk energy density predictions (line) and input data (circles) as a function of the relative time into lactation (% of the total duration) for species in the family Bovidae. Measurements that occurred <3% of the time into the lactation interval were not included. Subplots correspond to each species included in the analysis. The size of individual data points corresponds to the number of individual measurements associated with it, with the smallest size corresponding to a single measurement per point.
(TIFF)

S11 Fig Milk energy density predictions (line) and input data (circles) as a function of the relative time into lactation (% of the total duration) for species in the family Camelidae. Measurements that occurred <3% of the time into the lactation interval were not included. Subplots correspond to each species included in the analysis. The size of individual data points corresponds to the number of individual measurements associated with it, with the smallest size corresponding to a single measurement per point.
(TIFF)

S12 Fig Milk energy density predictions (line) and input data (circles) as a function of the relative time into lactation (% of the total duration) for species in the family Elephantidae. Measurements that occurred <3% of the time into the lactation interval were not included. Subplots correspond to each species included in the analysis. The size of individual data points corresponds to the number of individual measurements associated with it, with the smallest size corresponding to a single measurement per point.
(TIFF)

S13 Fig Milk energy density predictions (line) and input data (circles) as a function of the relative time into lactation (% of the total duration) for species in the family Cervidae. Measurements that occurred <3% of the time into the lactation interval were not included. Subplots correspond to each species included in the analysis. The size of individual data points corresponds to the number of individual measurements associated with it, with the smallest size corresponding to a single measurement per point.
(TIFF)

S14 Fig Milk energy density predictions (line) and input data (circles) as a function of the relative time into lactation (% of the total duration) for species in the families Delphinidae and Phocoenidae. Measurements that occurred <3% of the time into the lactation interval were not included. Subplots correspond to each species included in the analysis. The size of individual data points corresponds to the number of individual measurements associated with it, with the smallest size corresponding to a single measurement per point.
(TIFF)

S1 Table Milk intake rates (g day^-1^) and associated data on offspring, such as mass (g), age (days), and lactation duration (days). When available and necessary (N > 1), variability in milk intake rates and mass are included as well. Information associated with the data source and extraction methods are also shown. Measurements that occurred <3% of the time into the lactation interval were not used in the analysis. Data provided by study authors are shown only as summaries, with associated references and contact information.
(XLSX)

S2 Table Milk energy density (kJ g^-1^) and associated data on offspring, such as age (days) and lactation duration (days). When available and necessary (N > 1), variability in milk energy density is included as well. Information associated with the data source and extraction methods are also shown. Measurements that occurred <3% of the time into the lactation interval were not used in the analysis. Data provided by study authors are shown only as summaries, with associated references and contact information.
(XLSX)

S3 Table Mass-specific milk intake rate predictions (g day^-1^ g offspring^-0.82^) for each species included in the analysis. Predictions and associated lower and upper confidence intervals are provided as a proportion of time into lactation for each replicate dataset (Population) when it was necessary to simulate data, as described in the main text.
(ZIP)

S4 Table Milk energy density predictions (kJ g^-1^) for each species included in the analysis belonging to the Order Artiodactyla or Proboscidea. Predictions and associated lower and upper confidence intervals are provided as a proportion of time into lactation for each replicate dataset (Population) when it was necessary to simulate data, as described in the main text.
(ZIP)

S5 Table Milk energy density predictions (kJ g^-1^) for each species included in the analysis belonging to the Order Carnivora. Predictions and associated lower and upper confidence intervals are provided as a proportion of time into lactation for each replicate dataset (Population) when it was necessary to simulate data, as described in the main text.
(ZIP)
