# Supplementary figures and images for: Predicting daily lactation costs of marine mammals for use in bioenergetic models
Source: PLoS One. 2026 Jul 29;21(7):e0352443. doi: 10.1371/journal.pone.0352443 (PMC13419228; doi:10.1371/journal.pone.0352443)

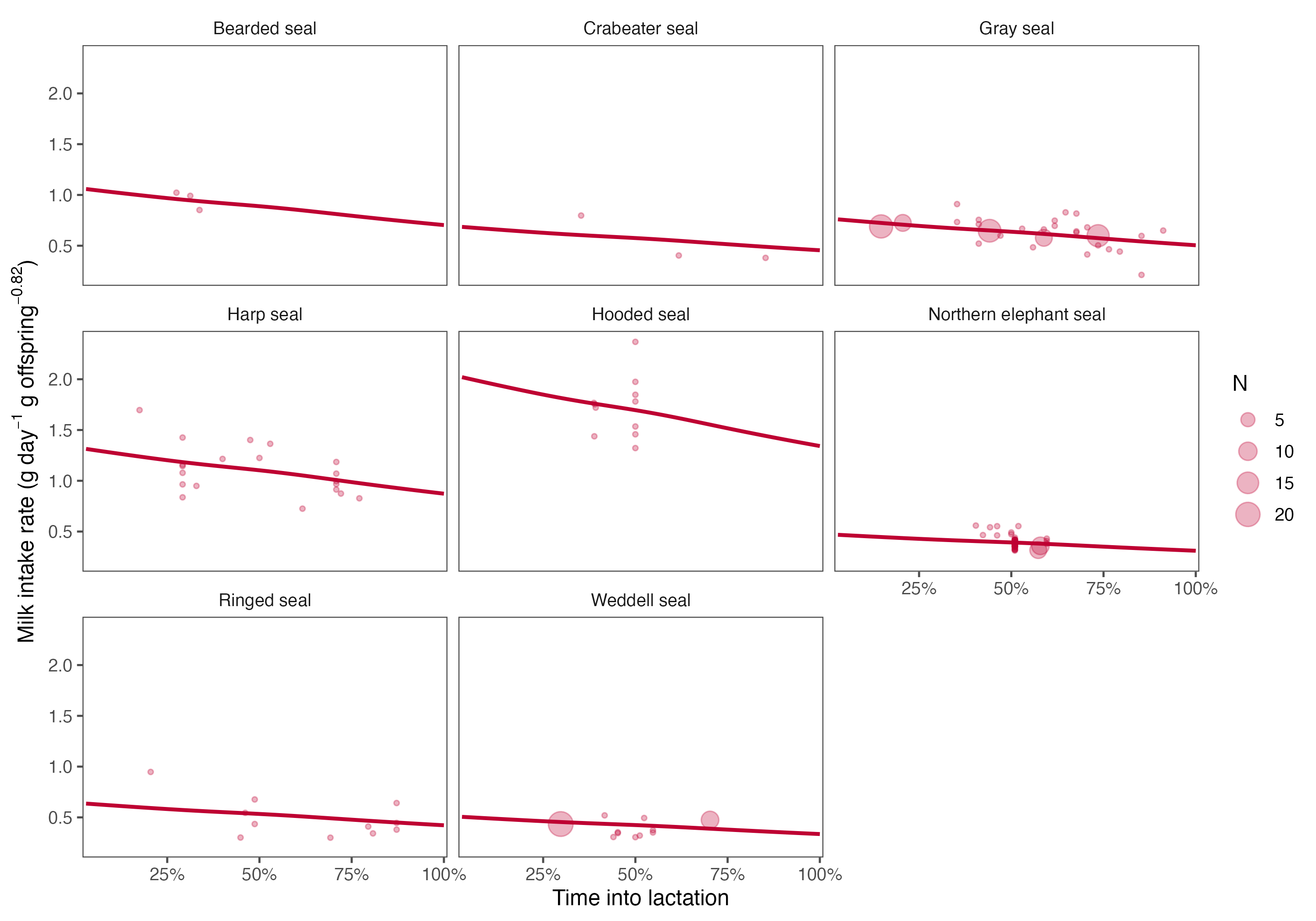

Supplement: S1 Fig — Measurements that occurred <3% of the time into the lactation interval were not included. Subplots correspond to each species included in the analysis. The size of individual data points corresponds to the number of individual measurements associated with it, with the smallest size corresponding to a single measurement per point. (TIFF) [file pone.0352443.s001.tiff]

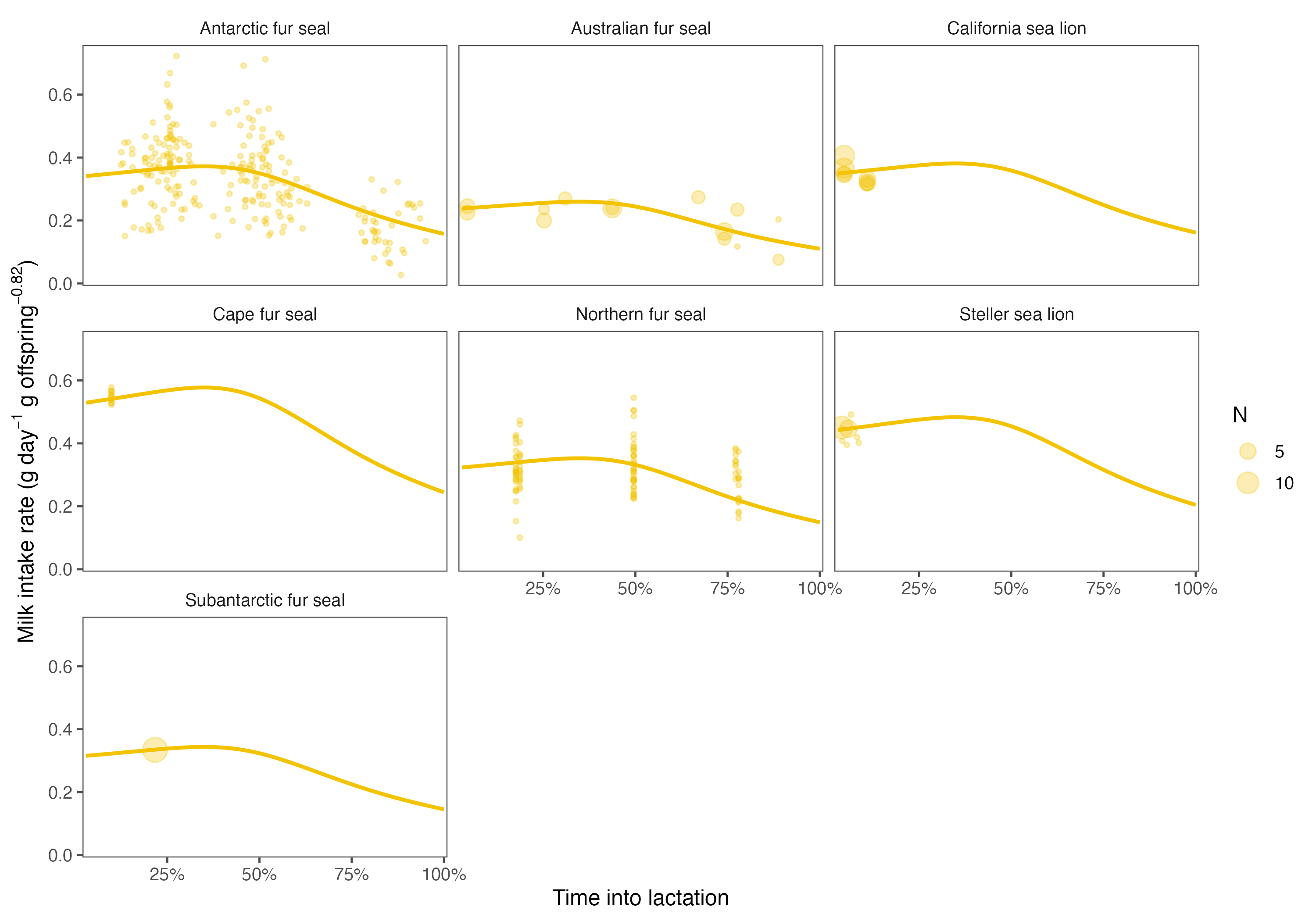

Supplement: S2 Fig — Measurements that occurred <3% of the time into the lactation interval were not included. Subplots correspond to each species included in the analysis. The size of individual data points corresponds to the number of individual measurements associated with it, with the smallest size corresponding to a single measurement per point. (TIFF) [file pone.0352443.s002.tiff]

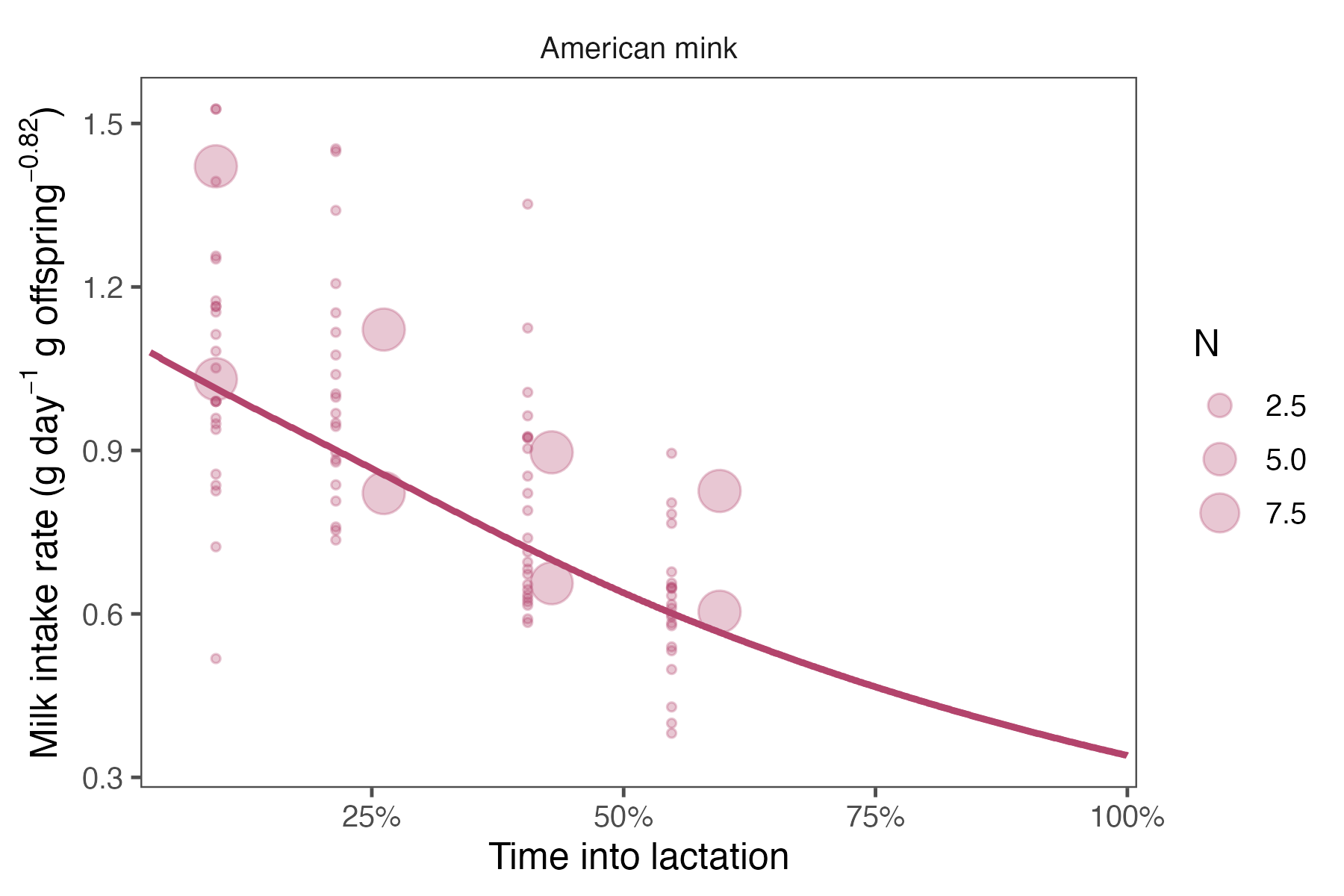

Supplement: S3 Fig — Measurements that occurred <3% of the time into the lactation interval were not included. The size of individual data points corresponds to the number of individual measurements associated with it, with the smallest size corresponding to a single measurement per point. (TIFF) [file pone.0352443.s003.tiff]

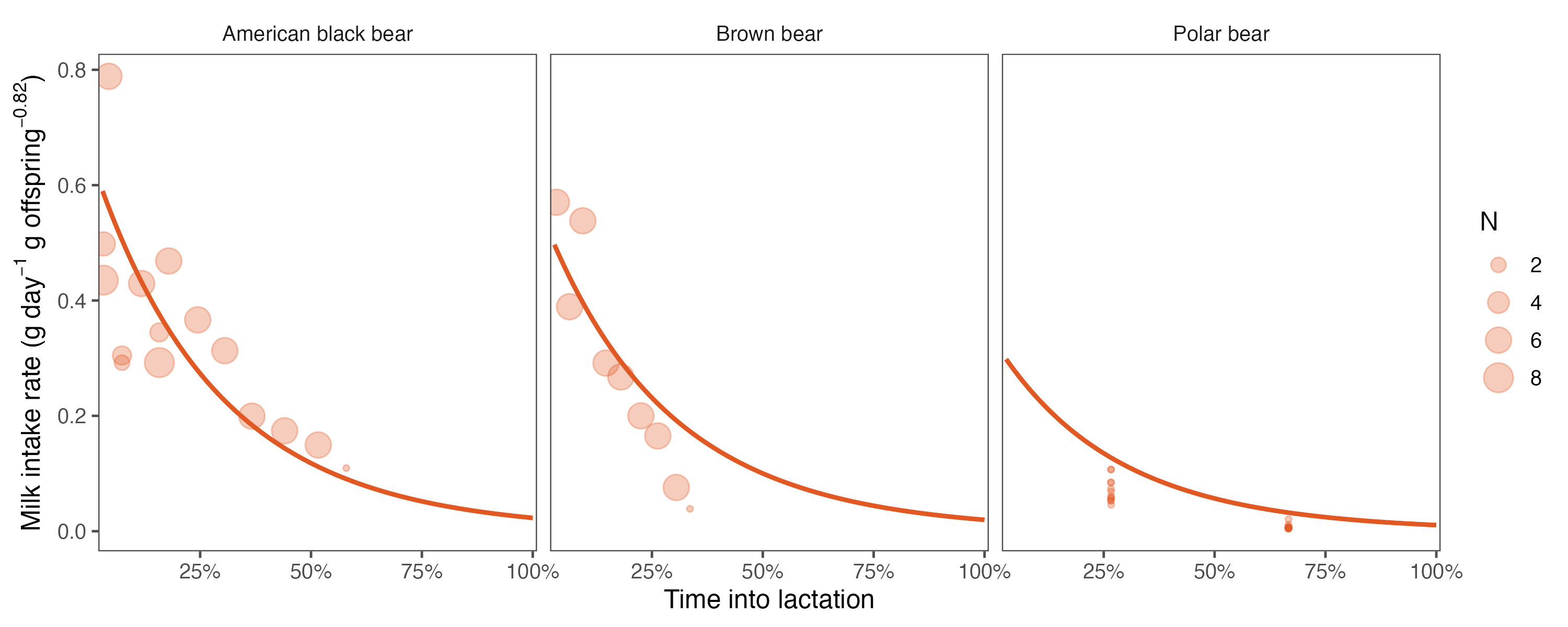

Supplement: S4 Fig — Measurements that occurred <3% of the time into the lactation interval were not included. Subplots correspond to each species included in the analysis. The size of individual data points corresponds to the number of individual measurements associated with it, with the smallest size corresponding to a single measurement per point. (TIFF) [file pone.0352443.s004.tiff]

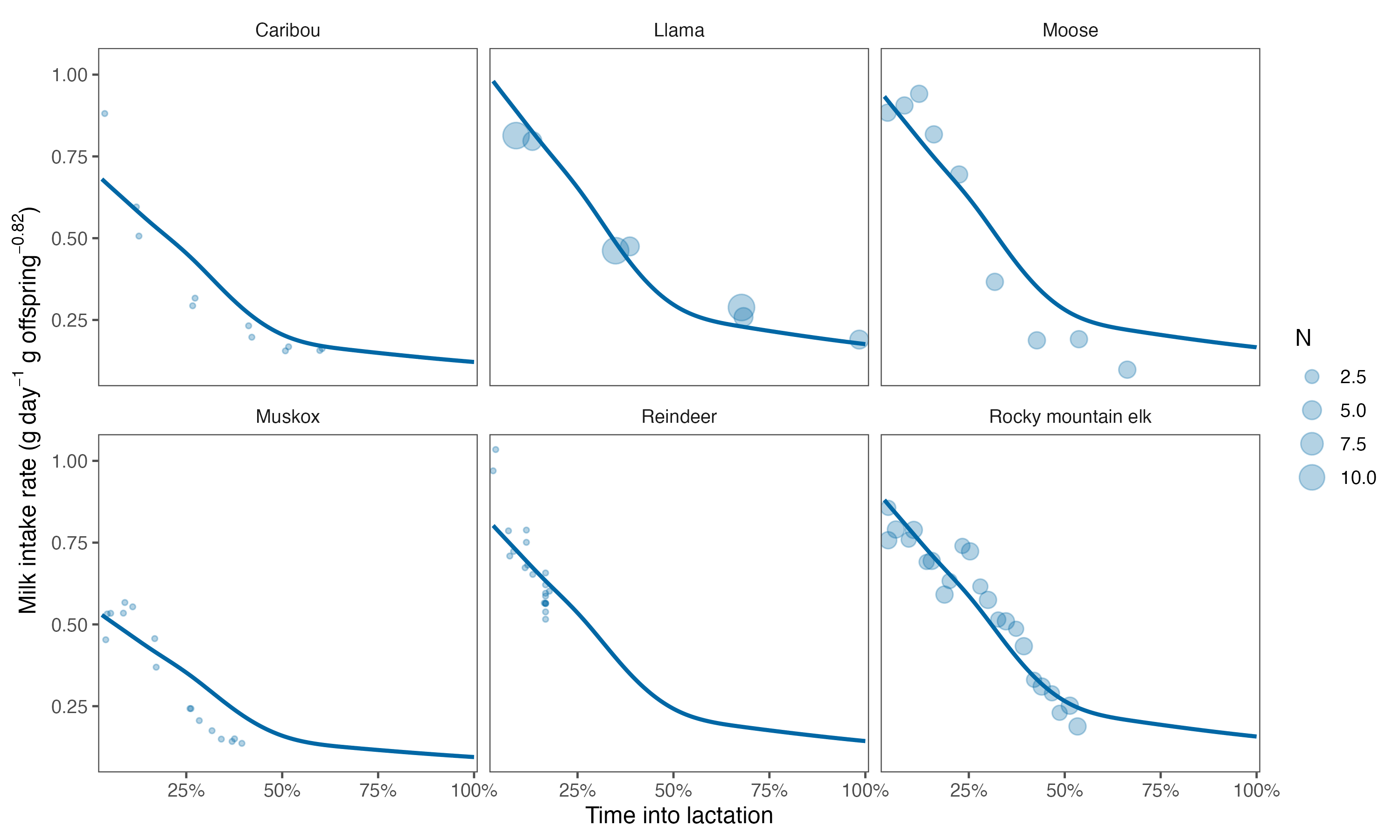

Supplement: S5 Fig — Measurements that occurred <3% of the time into the lactation interval were not included. Subplots correspond to each species included in the analysis. The size of individual data points corresponds to the number of individual measurements associated with it, with the smallest size corresponding to a single measurement per point. (TIFF) [file pone.0352443.s005.tiff]

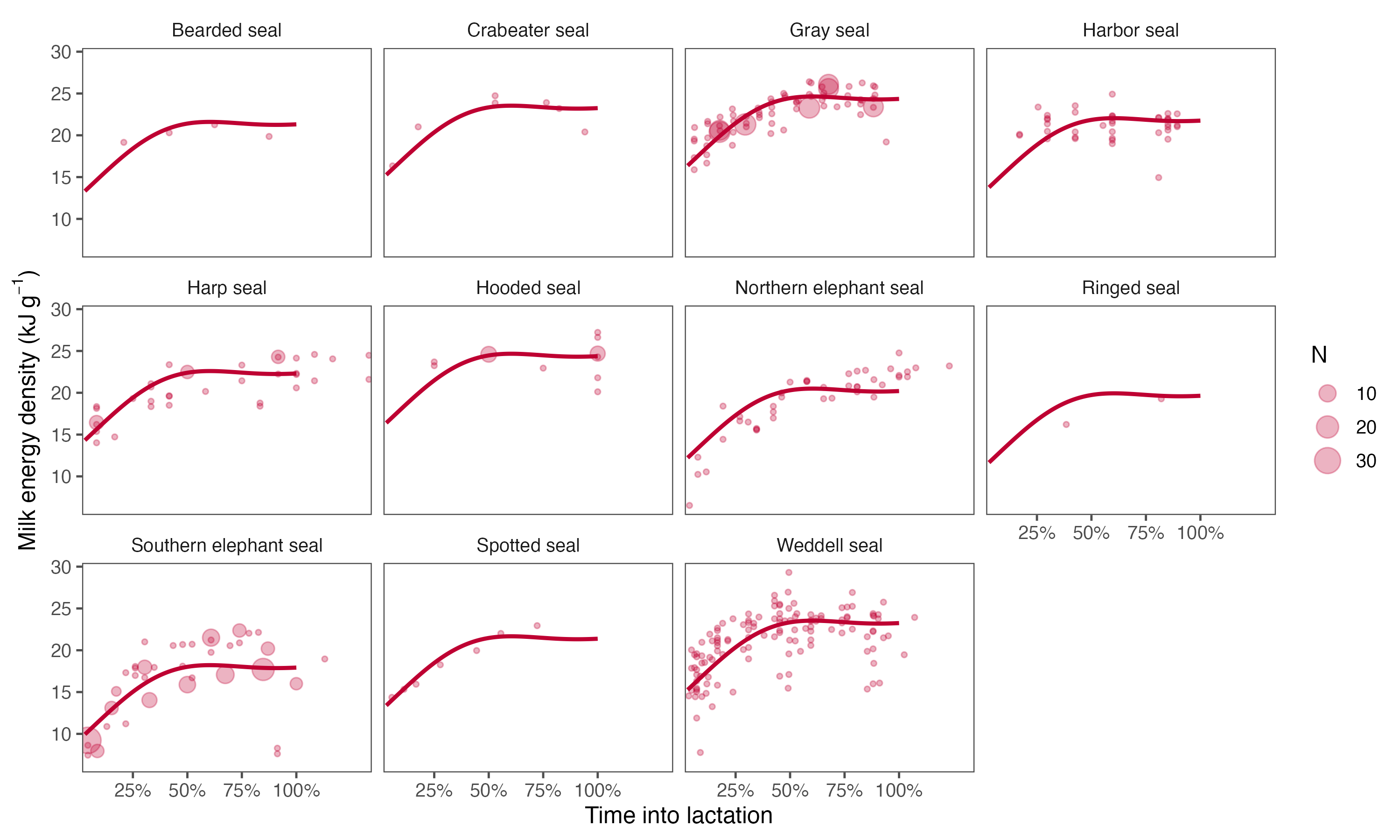

Supplement: S6 Fig — Measurements that occurred <3% of the time into the lactation interval were not included. Subplots correspond to each species included in the analysis. The size of individual data points corresponds to the number of individual measurements associated with it, with the smallest size corresponding to a single measurement per point. (TIFF) [file pone.0352443.s006.tiff]

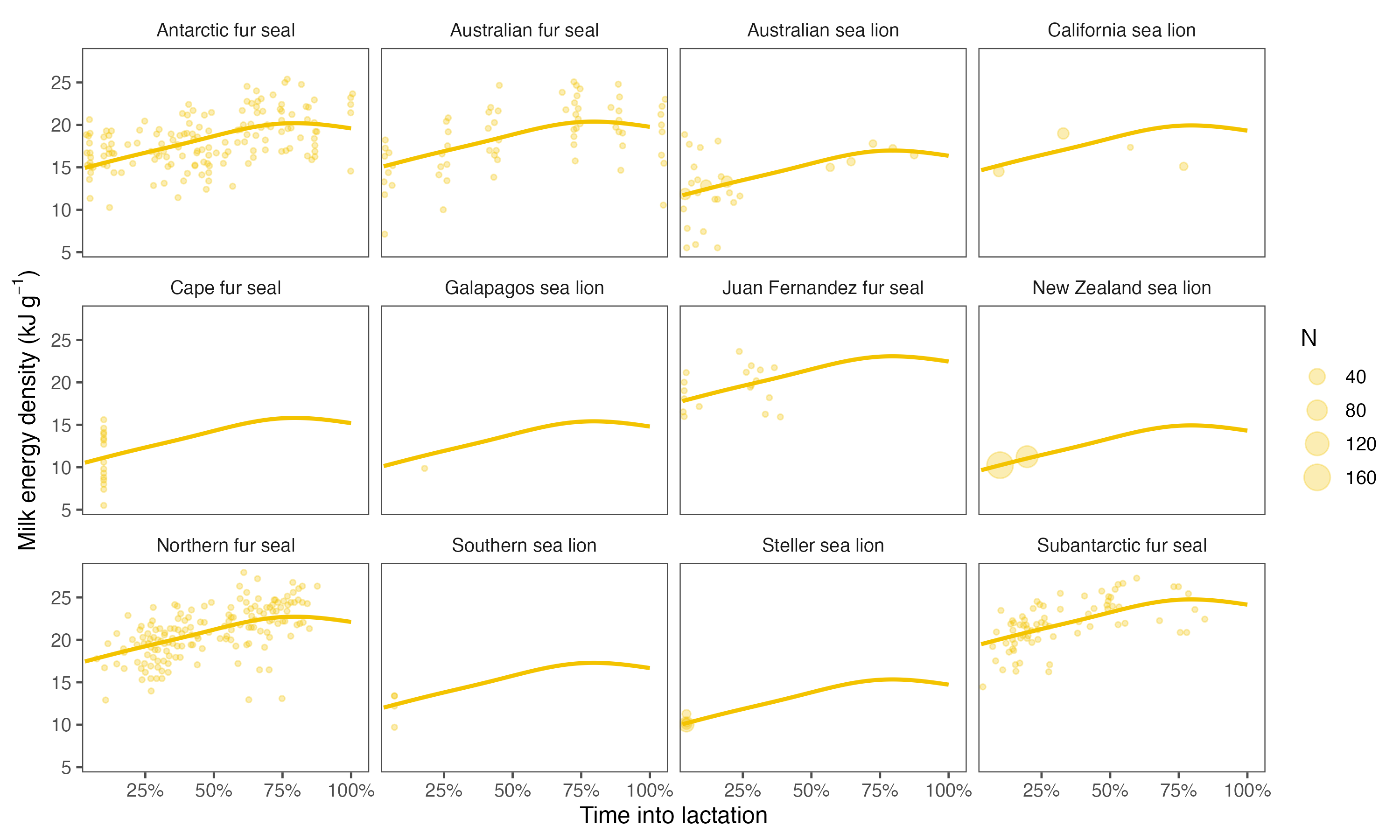

Supplement: S7 Fig — Measurements that occurred <3% of the time into the lactation interval were not included. Subplots correspond to each species included in the analysis. The size of individual data points corresponds to the number of individual measurements associated with it, with the smallest size corresponding to a single measurement per point. (TIFF) [file pone.0352443.s007.tiff]

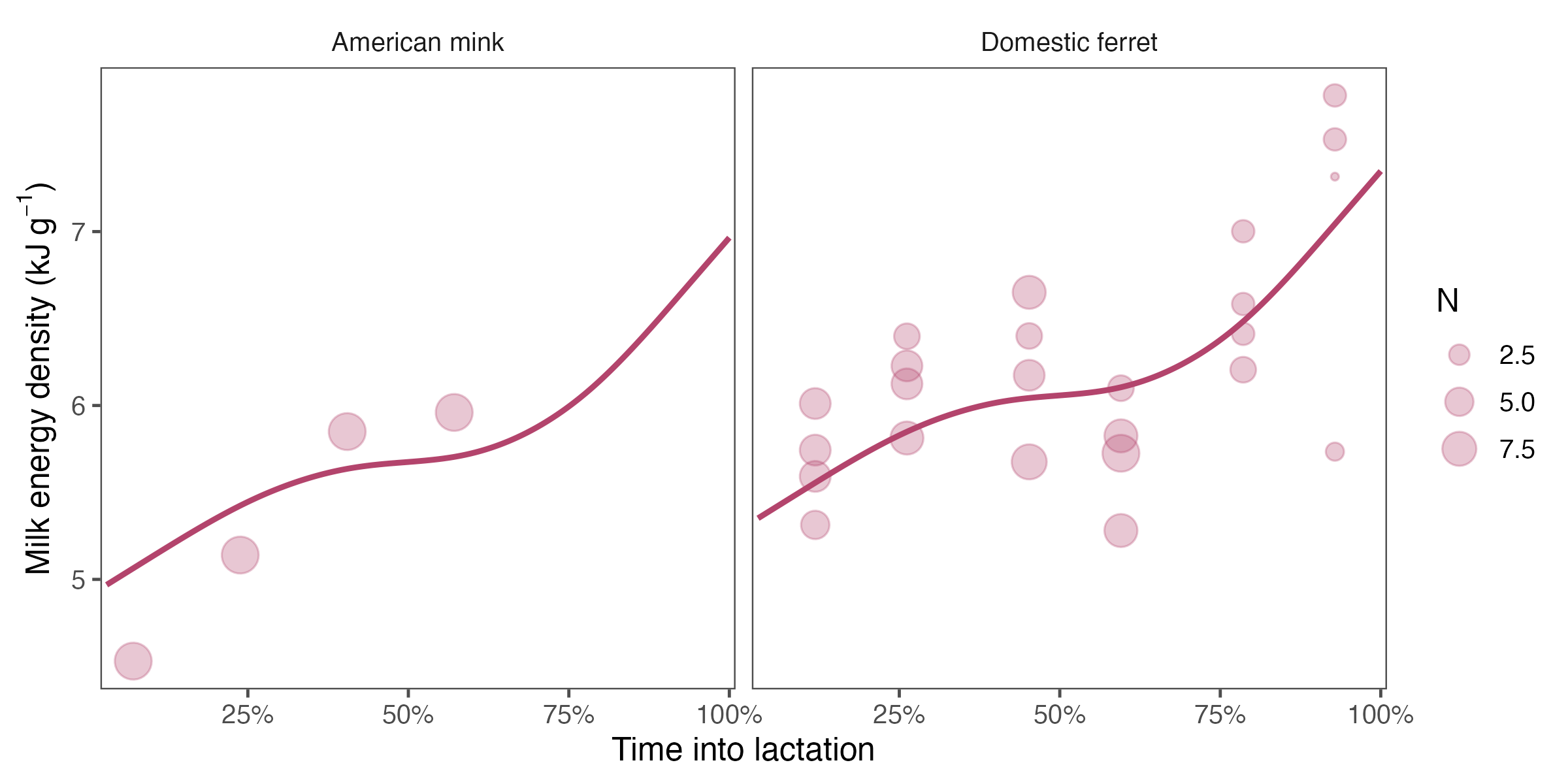

Supplement: S8 Fig — Measurements that occurred <3% of the time into the lactation interval were not included. Subplots correspond to each species included in the analysis. The size of individual data points corresponds to the number of individual measurements associated with it, with the smallest size corresponding to a single measurement per point. (TIFF) [file pone.0352443.s008.tiff]

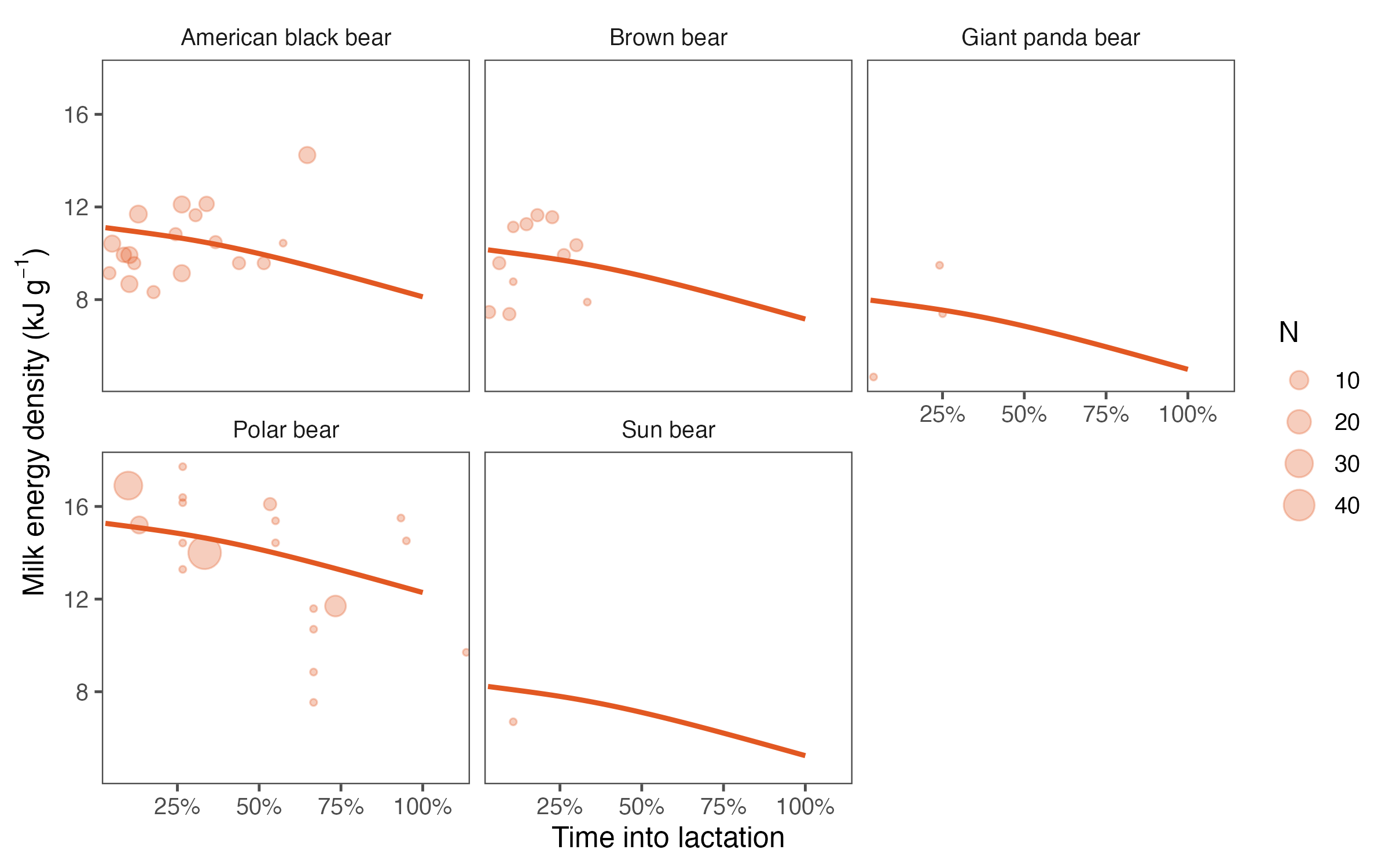

Supplement: S9 Fig — Measurements that occurred <3% of the time into the lactation interval were not included. Subplots correspond to each species included in the analysis. The size of individual data points corresponds to the number of individual measurements associated with it, with the smallest size corresponding to a single measurement per point. (TIFF) [file pone.0352443.s009.tiff]

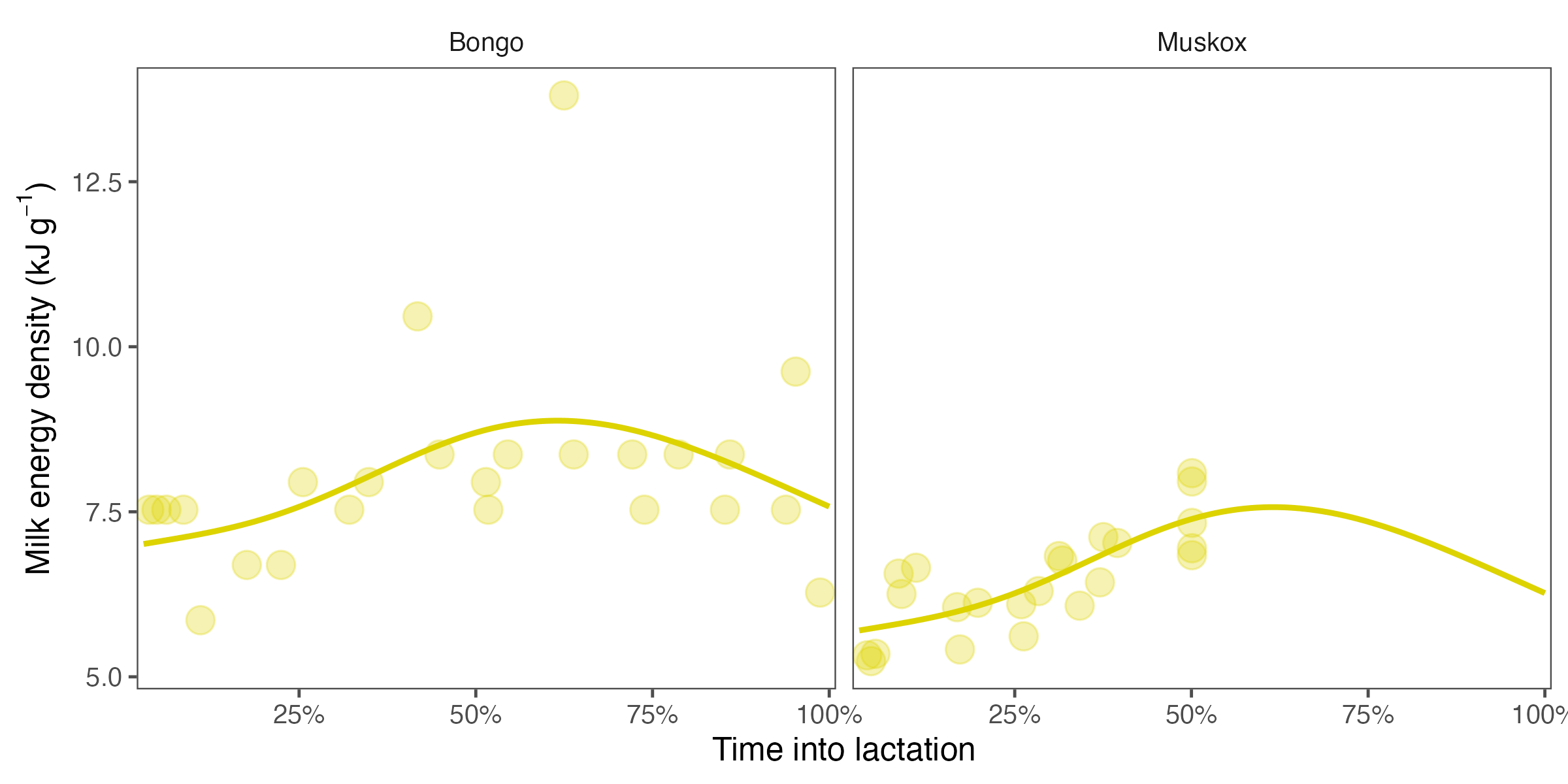

Supplement: S10 Fig — Measurements that occurred <3% of the time into the lactation interval were not included. Subplots correspond to each species included in the analysis. The size of individual data points corresponds to the number of individual measurements associated with it, with the smallest size corresponding to a single measurement per point. (TIFF) [file pone.0352443.s010.tiff]

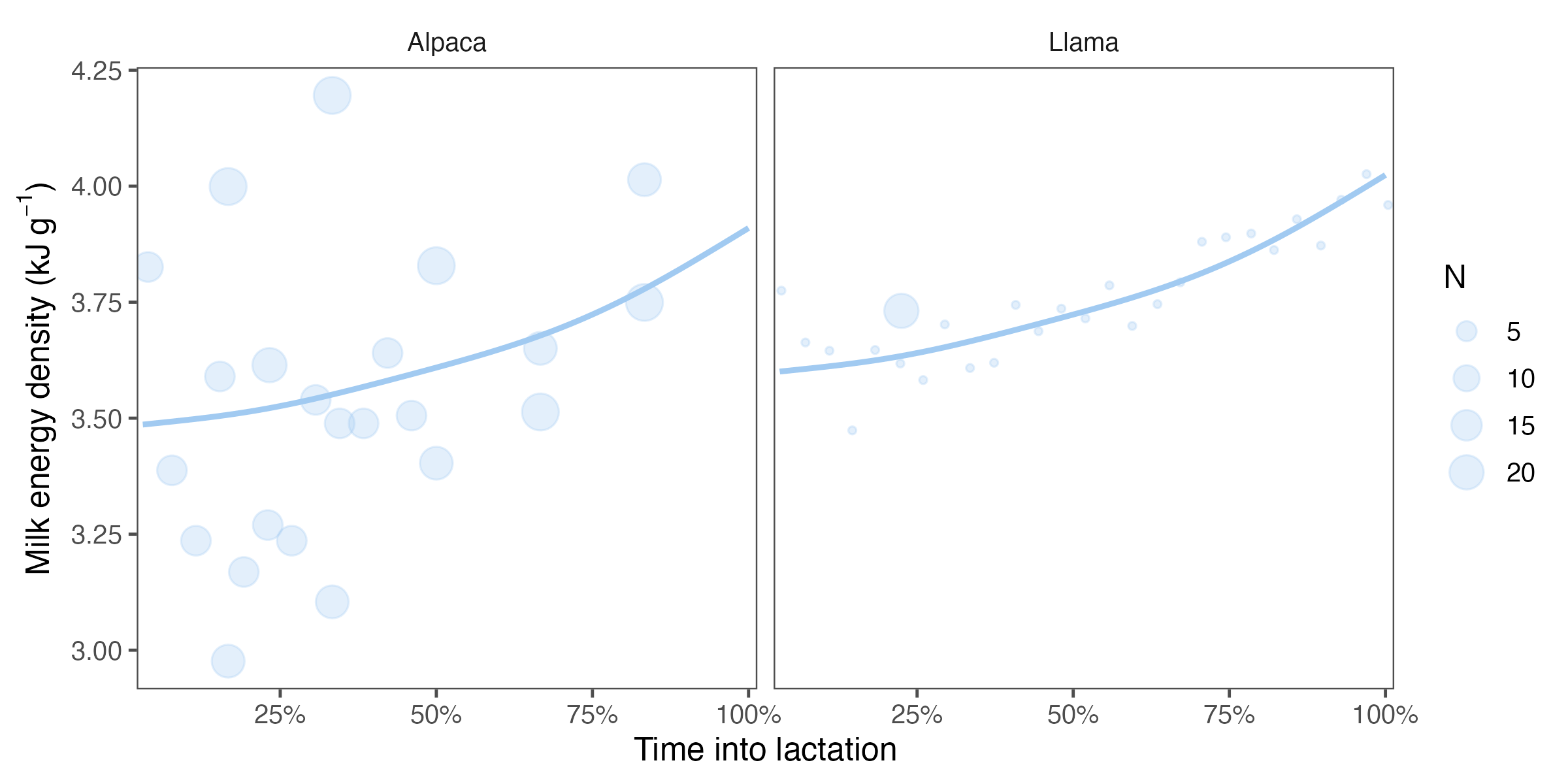

Supplement: S11 Fig — Measurements that occurred <3% of the time into the lactation interval were not included. Subplots correspond to each species included in the analysis. The size of individual data points corresponds to the number of individual measurements associated with it, with the smallest size corresponding to a single measurement per point. (TIFF) [file pone.0352443.s011.tiff]

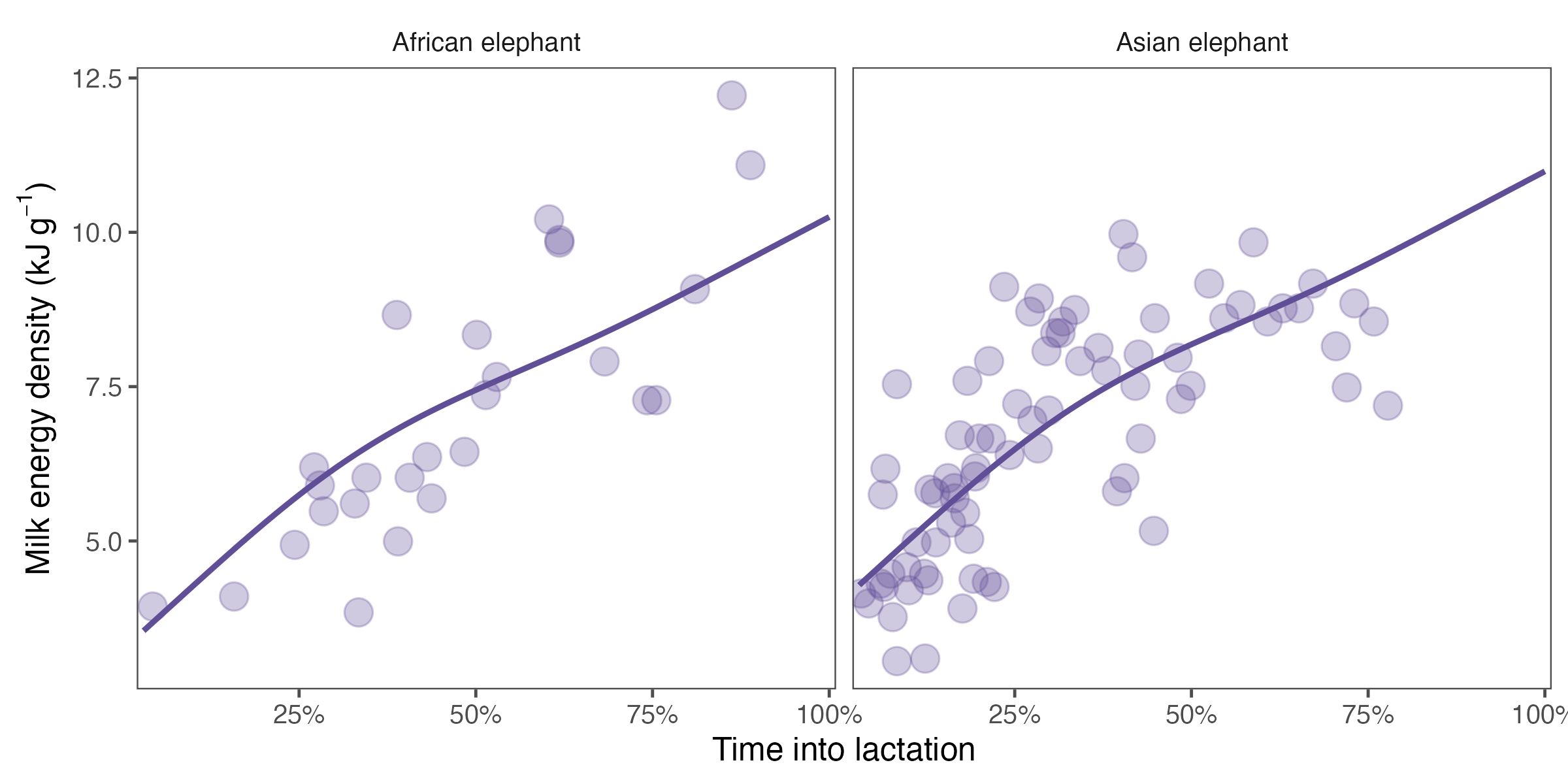

Supplement: S12 Fig — Measurements that occurred <3% of the time into the lactation interval were not included. Subplots correspond to each species included in the analysis. The size of individual data points corresponds to the number of individual measurements associated with it, with the smallest size corresponding to a single measurement per point. (TIFF) [file pone.0352443.s012.tiff]

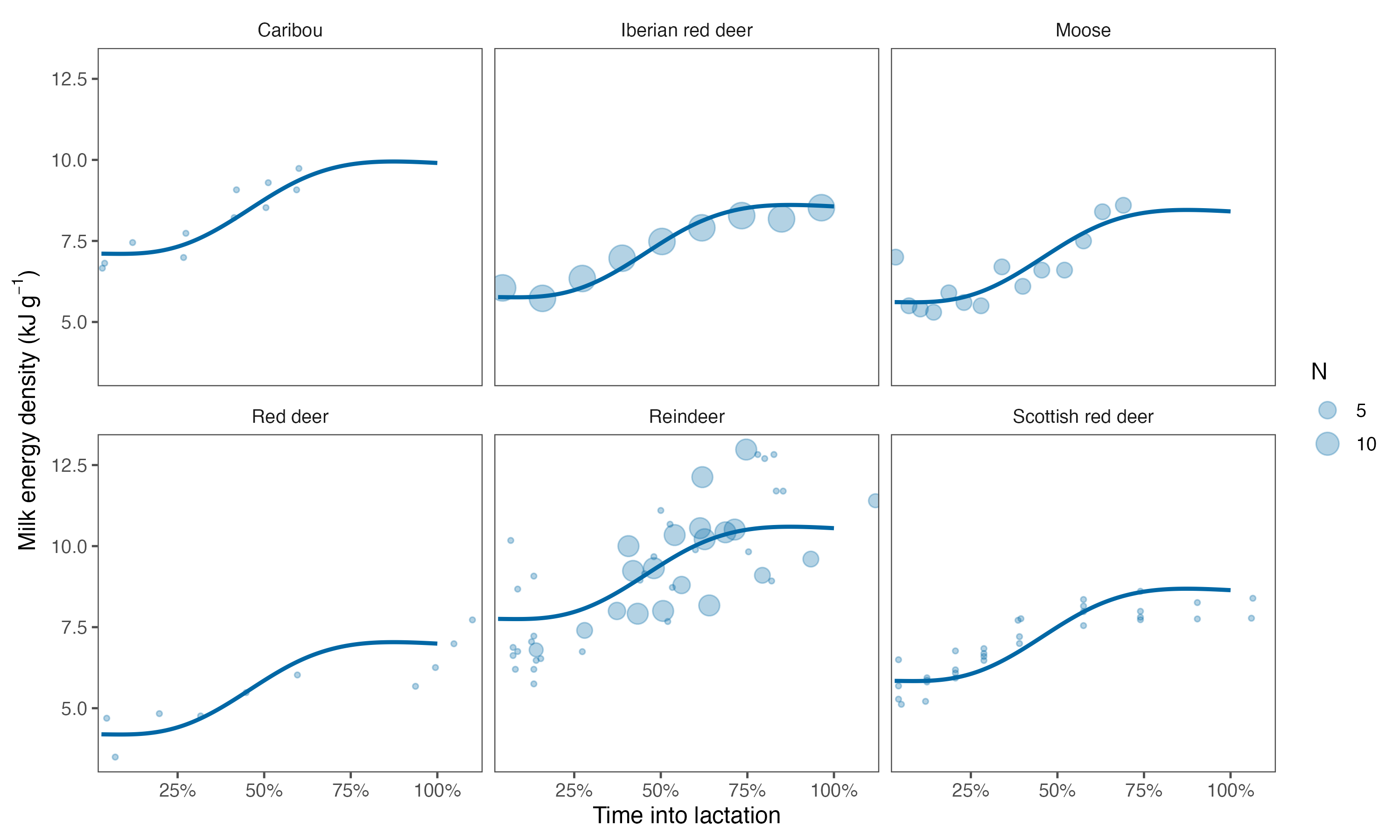

Supplement: S13 Fig — Measurements that occurred <3% of the time into the lactation interval were not included. Subplots correspond to each species included in the analysis. The size of individual data points corresponds to the number of individual measurements associated with it, with the smallest size corresponding to a single measurement per point. (TIFF) [file pone.0352443.s013.tiff]

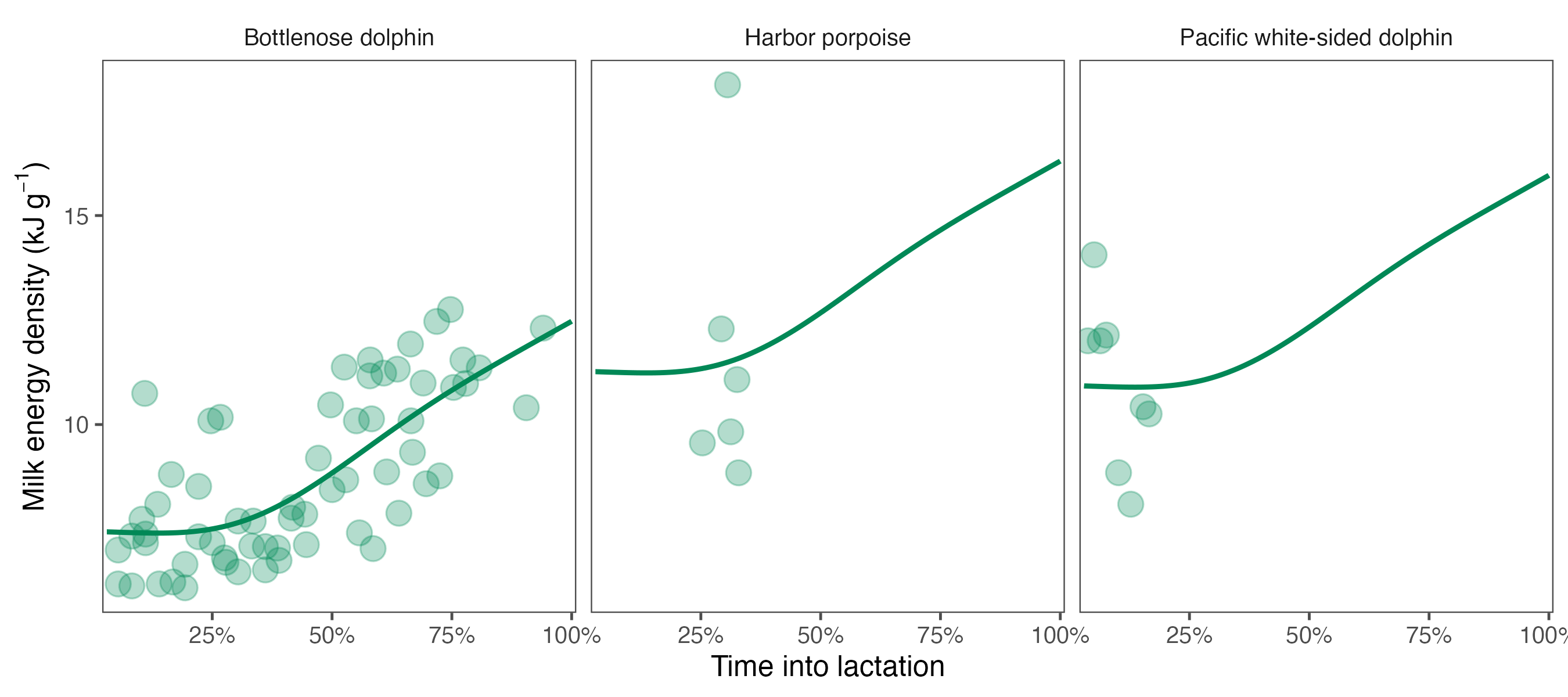

Supplement: S14 Fig — Measurements that occurred <3% of the time into the lactation interval were not included. Subplots correspond to each species included in the analysis. The size of individual data points corresponds to the number of individual measurements associated with it, with the smallest size corresponding to a single measurement per point. (TIFF) [file pone.0352443.s014.tiff]
