# Supplementary material for: Predicting daily lactation costs of marine mammals for use in bioenergetic models
Source: PLoS One. 2026 Jul 29;21(7):e0352443. doi: 10.1371/journal.pone.0352443 (PMC13419228; doi:10.1371/journal.pone.0352443)
Supplement: Peer review history

# Peer review: Decision Letter 0

**DOI:** 10.1371/journal.pone.0352443.r001

**Authors:** Nathan Wolf

19 Apr 2026

PONE-D-25-43306

Predicting daily lactation costs of marine mammals for use in bioenergetic models

PLOS One

Dear Dr. McHuron,

Thank you for submitting your manuscript to PLOS ONE. After careful consideration, we feel that it has merit but does not fully meet PLOS ONE’s publication criteria as it currently stands. Therefore, we invite you to submit a revised version of the manuscript that addresses the points raised during the review process.

Please submit your revised manuscript by Jun 03 2026 11:59PM. If you will need more time than this to complete your revisions, please reply to this message or contact the journal office at plosone@plos.org. When you're ready to submit your revision, log on to https://www.editorialmanager.com/pone/ and select the 'Submissions Needing Revision' folder to locate your manuscript file.

Please include the following items when submitting your revised manuscript:

- A letter that responds to each point raised by the academic editor and reviewer(s). You should upload this letter as a separate file labeled 'Response to Reviewers'.
- A marked-up copy of your manuscript that highlights changes made to the original version. You should upload this as a separate file labeled 'Revised Manuscript with Track Changes'.
- An unmarked version of your revised paper without tracked changes. You should upload this as a separate file labeled 'Manuscript'.

If you would like to make changes to your financial disclosure, please include your updated statement in your cover letter. Guidelines for resubmitting your figure files are available below the reviewer comments at the end of this letter.

If applicable, we recommend that you deposit your laboratory protocols in protocols.io to enhance the reproducibility of your results. Protocols.io assigns your protocol its own identifier (DOI) so that it can be cited independently in the future. For instructions see: https://journals.plos.org/plosone/s/submission-guidelines#loc-laboratory-protocols. Additionally, PLOS ONE offers an option for publishing peer-reviewed Lab Protocol articles, which describe protocols hosted on protocols.io. Read more information on sharing protocols at https://plos.org/protocols?utm_medium=editorial-email&utm_source=authorletters&utm_campaign=protocols.

As the corresponding author, your ORCID iD is verified in the submission system and will appear in the published article. PLOS supports the use of ORCID, and we encourage all coauthors to register for an ORCID iD and use it as well. Please encourage your coauthors to verify their ORCID iD within the submission system before final acceptance, as unverified ORCID iDs will not appear in the published article. *Only*  the individual author can complete the verification step; PLOS staff *cannot*  verify ORCID iDs on behalf of authors.

We look forward to receiving your revised manuscript.

Kind regards,

Nathan Wolf

Academic Editor

PLOS One

Journal Requirements:

When submitting your revision, we need you to address these additional requirements.

1.Please ensure that your manuscript meets PLOS ONE's style requirements, including those for file naming. The PLOS ONE style templates can be found at

https://journals.plos.org/plosone/s/file?id=wjVg/PLOSOne_formatting_sample_main_body.pdf and

https://journals.plos.org/plosone/s/file?id=ba62/PLOSOne_formatting_sample_title_authors_affiliations.pdf

2. Please note that PLOS One has specific guidelines on code sharing for submissions in which author-generated code underpins the findings in the manuscript. In these cases, we expect all author-generated code to be made available without restrictions upon publication of the work. Please review our guidelines at https://journals.plos.org/plosone/s/materials-and-software-sharing#loc-sharing-code and ensure that your code is shared in a way that follows best practice and facilitates reproducibility and reuse.

3. Thank you for stating the following financial disclosure:

“This study was funded under Office of Naval Research grant number N000142312492.”

Please state what role the funders took in the study.  If the funders had no role, please state: "The funders had no role in study design, data collection and analysis, decision to publish, or preparation of the manuscript."

If this statement is not correct you must amend it as needed.

Please include this amended Role of Funder statement in your cover letter; we will change the online submission form on your behalf.

4. We are unable to open your Supporting Information file S3_Table.gz, S4_Table.gz and S5_Table.gz. Please kindly revise as necessary and re-upload.

5.If the reviewer comments include a recommendation to cite specific previously published works, please review and evaluate these publications to determine whether they are relevant and should be cited. There is no requirement to cite these works unless the editor has indicated otherwise.

6. Please review your reference list to ensure that it is complete and correct. If you have cited papers that have been retracted, please include the rationale for doing so in the manuscript text, or remove these references and replace them with relevant current references. Any changes to the reference list should be mentioned in the rebuttal letter that accompanies your revised manuscript. If you need to cite a retracted article, indicate the article’s retracted status in the References list and also include a citation and full reference for the retraction notice.

Additional Editor Comments:

Thank you for your extraordinary patience during the review process. As I mentioned in our correspondence, it was difficult to find qualified reviewers for this manuscript. That being said, both Reviewers were extremely enthusiastic about the work. Each recommended several minor edits, which I agree will strengthen the manuscript. I look forward to reading a revised version.

[Note: HTML markup is below. Please do not edit.]

Reviewers' comments:

Reviewer's Responses to Questions

**Comments to the Author**

1. Is the manuscript technically sound, and do the data support the conclusions?

The manuscript must describe a technically sound piece of scientific research with data that supports the conclusions. Experiments must have been conducted rigorously, with appropriate controls, replication, and sample sizes. The conclusions must be drawn appropriately based on the data presented.

Reviewer #1: Yes

Reviewer #2: Yes

**********

2. Has the statistical analysis been performed appropriately and rigorously?

Reviewer #1: Yes

Reviewer #2: Yes

**********

3. Have the authors made all data underlying the findings in their manuscript fully available?

The PLOS Data policy requires authors to make all data underlying the findings described in their manuscript fully available without restriction, with rare exception (please refer to the Data Availability Statement in the manuscript PDF file). The data should be provided as part of the manuscript or its supporting information, or deposited to a public repository. For example, in addition to summary statistics, the data points behind means, medians and variance measures should be available. If there are restrictions on publicly sharing data—e.g. participant privacy or use of data from a third party—those must be specified.

Reviewer #1: Yes

Reviewer #2: Yes

**********

4. Is the manuscript presented in an intelligible fashion and written in standard English?

PLOS ONE does not copyedit accepted manuscripts, so the language in submitted articles must be clear, correct, and unambiguous. Any typographical or grammatical errors should be corrected at revision, so please note any specific errors here.

Reviewer #1: Yes

Reviewer #2: Yes

**********

5. Review Comments to the Author

Please use the space provided to explain your answers to the questions above. You may also include additional comments for the author, including concerns about dual publication, research ethics, or publication ethics. (Please upload your review as an attachment if it exceeds 20,000 characters)

Reviewer #1: General comments

Thanks for the opportunity to review this interesting and important study, where the author reviews the available data/information on milk intake rates and milk energy density over the course of lactation for a range of species, to investigate whether predictions from data-rich species can inform estimates for poorly studied ones. The datasets that were compiled and the associated analyses represent a useful resource for marine mammal bioenergetic studies, and the validation of predictions using independent estimates from poorly represented taxa is extremely informative. Therefore, the study provides both a useful methodology to derive appropriate values for some species where predictions from related taxa are relevant, and a roadmap for future research in other species where these predictions appear to perform poorly.

Overall, I have no major concerns with the paper. The methods described for collating information from the literature and for analysing the resulting datasets are appropriate and clearly presented. However, I found that some important aspects of the ecology of marine mammals that likely affect lactation patterns, while being mentioned, were not explored as I would have expected; for example, the distinction between capital and income breeding strategies. I wonder if this warrants a more prominent discussion, given the strong influence breeding strategy will have on milk intake rates and energy density – has the author considered an analysis based on ecological similarity, rather than taxonomy? This could complement the family/order-level analysis, especially because the latter does not allow for species-level differences (and important variation may therefore be masked in the results). Other important aspects, such as the role of the gradual transition to independent feeding (particularly in species with extended lactation times, e.g. odontocetes), or the large variation/plasticity in lactation duration are indeed highlighted at various points in the Discussion, but I was left wondering what influence some of those simplifying assumptions could have on the results and conclusions drawn. There might be scope for some further reflection on those points.

From a stylistic perspective, the paper is very well written, and the presentation of the methodology and results is effective and concise. Congratulations to the author for this timely study, which will provide a critical reference for marine mammal bioenergetic modelling going forward.

Detailed comments

Line 20: I’m not sure you need to report the units in the Abstract. This could just be rephrased to ‘rates of milk intake per unit of offspring mass’, or some such.

Line 27: ‘independent estimates of THESE COSTS FOR cetaceans’, or ‘of these parameters’; ‘estimates of cetaceans’ doesn’t really make sense.

Line 62: perhaps change to ‘in comparisons involving..’?

Line 81: “opportunistic collection… HAS provided”.

Line 88: is the transfer of milk from the mother’s mammary gland to the offspring 100% efficient? The statements at lines 216 and 259 seem in contradiction with this. Also, does the energy that the mother invests in producing milk convert exactly to the energy contained in the milk? In general, I find this interchangeable terminology potentially confusing (and inappropriate, if there are inefficiencies); I would choose one of the two terms based on what is actually captured in these analyses, and stick with it throughout.

Lines 116-119: do you expect this methodological variation to impact the results in any relevant way?

Line 135-138: just a note to underline that, while I understand the rationale for this necessary choice, this is a major simplification. In some species, the variation in lactation duration is large and, in fact, lactation duration is plastically adjusted depending on external conditions and, probably, condition of the mother and the offspring. This would be fine if sample sizes were large, but because the numbers are small, the specific animals that were sampled could have a large influence on the results. This is highlighted in the Discussion, but there could be some further reflection on its potential influence on the conclusions.

Line 145: if these data were compiled, it might be worth including them in a separate supplementary table anyway (even if they are not used in the analysis); having a record of published estimates, with rough lactation stage, could be a useful resource for other studies.

Line 149: if I understand correctly, the models described in the next paragraphs were repeated 50 times (using the 50 simulated datasets), right? It might be worth stating this explicitly, and describing how the results from the 50 models were then combined when presenting the results.

Line 154: I would spell out the rationale for using offspring-0.83 as the unit.

Line 159: “data… WERE”.

Line 161: I would clarify that the random effect is on the intercept, and what that means in practice. On a related note: how reasonable is it to assume a single spline across all species in these diverse families (particularly for the order artiodactyls)? Could this contribute to the poor predictive power of the estimated relationships for cetaceans? I understand the rationale of this decision (line 166), and the caveat is highlighted in the Discussion, but this may require further reflection. The results (lines 286-287) also suggest that this assumption is likely too simplistic.

Line 162: Fixing k to avoid overfitting is controversial. While commonly used, it is not best practice. A better way to impose smoother relationships may be to increase the penalty for wiggliness using the gamma argument. I suspect it’ll make little difference in this case, though.

Line 178: Referring to these estimates as ‘data’ and ‘empirical estimates’ doesn’t sit well with me, for some of those cases. Some estimates are non-validated calculations largely based on strong assumptions around the value of many bioenergetic/morphometric parameters (e.g. for sea otters and the two cetaceans). It’s important to be clear that these are not direct measurements of milk intake or energy density. This caveat is spelled out below (199-202); still, I would avoid referring to these as ‘data’ or ‘empirical estimates’, and instead use ‘independent estimates’ throughout.

Line 270: Ditto. “The independent dataset was a bioenergetic model” is an oxymoron to me.

Line 298, Table 1: I would report the units in the column names.

Lines 306-311 and 316-320: these are notes for the Tables, correct? It seems like an unusual way to describe Table content.

Line 322 and 335: could you clarify what you mean by ‘conditional predictions’?

Line 335, Figure 2: I imagine that a plot reporting the underpinning data would be hard to read. However, I suggest including family-specific plots of the predictions and data points coloured by species in supplementary material, to help the reader assess the goodness-of-fit of those models visually.

Line 492: “aRe likely”.

Reviewer #2: This study represents a tremendous undertaking to leverage the use of existing data from marine and terrestrial mammals explore changes in milk intake rates and energy density throughout lactation, with a goal of developing predictive models that can be applied to bioenergetic models for data poor marine mammal species. There is a great need to explore that application, and this study handles complex and somewhat messy data in a comprehensive and organized way. The motivation and background for the study are clearly described, as are the methods including steps taken to convert data into consistent and comparable measures. Generalized GAMs are appropriate for handling these data. The use of independent data sources to explore model performance is a clever use of combining other data sources. The results are similarly clearly described. The discussion presented key findings that were supported by the analysis, and just as importantly, caveats and cautions of this analytical approach. While not the primary objective of this study, some taxonomic and life history comparisons were appropriately made to interpret findings. This was as rigorous an approach as could be supported by these datasets, and highlights areas where understanding is lacking and more research is needed. I though the manuscript overall was clear and flowed logically.

Specific Questions and Minor typos:

Keywords on the form reads “Milk intakel” but is correct in the manuscript so want to ensure the journal uses the correct one.

154: Is there an appropriate citation(s) for the use of -0.83 for scaling? Mostly for readers not immediately acquainted with metabolic scaling.

492: “ate” should be “are” in the middle of the sentence.

**********

6. PLOS authors have the option to publish the peer review history of their article (what does this mean?). If published, this will include your full peer review and any attached files.

If you choose “no”, your identity will remain anonymous but your review may still be made public.

**Do you want your identity to be public for this peer review?**  For information about this choice, including consent withdrawal, please see our Privacy Policy.

Reviewer #1: No

Reviewer #2: No

**********

[NOTE: If reviewer comments were submitted as an attachment file, they will be attached to this email and accessible via the submission site. Please log into your account, locate the manuscript record, and check for the action link "View Attachments". If this link does not appear, there are no attachment files.]

To ensure your figures meet our technical requirements, please review our figure guidelines: https://journals.plos.org/plosone/s/figures

You may also use PLOS’s free figure tool, NAAS, to help you prepare publication quality figures: https://journals.plos.org/plosone/s/figures#loc-tools-for-figure-preparation.

NAAS will assess whether your figures meet our technical requirements by comparing each figure against our figure specifications.

# Author comment: Author response to Decision Letter 1

**DOI:** 10.1371/journal.pone.0352443.r002

2 Jun 2026

These responses are also provided in the attached "Response to Reviewers" file. In addition, I have reviewed the references for correctness, made sure the formatting matches what is in the provided examples by the journal, and added to my data availability statement.

R:I appreciate the positive and helpful comments by both reviewers and the opportunity to submit a revised version of the manuscript. Below I have provided responses to each comment and descriptions of how the manuscript was revised to address those comments.

Reviewer #1: General comments

Thanks for the opportunity to review this interesting and important study, where the author reviews the available data/information on milk intake rates and milk energy density over the course of lactation for a range of species, to investigate whether predictions from data-rich species can inform estimates for poorly studied ones. The datasets that were compiled and the associated analyses represent a useful resource for marine mammal bioenergetic studies, and the validation of predictions using independent estimates from poorly represented taxa is extremely informative. Therefore, the study provides both a useful methodology to derive appropriate values for some species where predictions from related taxa are relevant, and a roadmap for future research in other species where these predictions appear to perform poorly.

Overall, I have no major concerns with the paper. The methods described for collating information from the literature and for analysing the resulting datasets are appropriate and clearly presented. However, I found that some important aspects of the ecology of marine mammals that likely affect lactation patterns, while being mentioned, were not explored as I would have expected; for example, the distinction between capital and income breeding strategies. I wonder if this warrants a more prominent discussion, given the strong influence breeding strategy will have on milk intake rates and energy density – has the author considered an analysis based on ecological similarity, rather than taxonomy? This could complement the family/order-level analysis, especially because the latter does not allow for species-level differences (and important variation may therefore be masked in the results). Other important aspects, such as the role of the gradual transition to independent feeding (particularly in species with extended lactation times, e.g. odontocetes), or the large variation/plasticity in lactation duration are indeed highlighted at various points in the Discussion, but I was left wondering what influence some of those simplifying assumptions could have on the results and conclusions drawn. There might be scope for some further reflection on those points.

From a stylistic perspective, the paper is very well written, and the presentation of the methodology and results is effective and concise. Congratulations to the author for this timely study, which will provide a critical reference for marine mammal bioenergetic modelling going forward.

R: I did initially consider and explore including some additional predictor variables in taxonomic-specific models, particularly for phocids and otariids, but in the end thought that factors would be too confounding among body size, lactation duration, and lactation strategy due to the limited number of species present in the analysis. If I had pooled across taxonomic groups, then I would have to contend with the addition of accounting for taxonomy and again thought that species representation may have been too limited to really draw any valuable conclusions. In regards to the discussion, I have included some additional mention of income vs capital breeding in the phocid paragraph, highlighting that it did not appear to be a neccesary metric for selecting a proxy species, at least relative to other factors like habitat type or lactation duration. I also specifically point out now that on a per nursing day, phocids and otariids appear to have similar milk intake rates despite using different lactation strategies. I didn’t want to add too much more to the discussion since I didn’t add anything to the analysis and I am hesitant to really say it is not important at all.

Detailed comments

Line 20: I’m not sure you need to report the units in the Abstract. This could just be rephrased to ‘rates of milk intake per unit of offspring mass’, or some such.

R: I revised to remove units and this now reads “Daily mass-specific milk intake rates”. I also removed the units from a later part of the abstract to be consistent with this revision.

Line 27: ‘independent estimates of THESE COSTS FOR cetaceans’, or ‘of these parameters’; ‘estimates of cetaceans’ doesn’t really make sense.

R: Changed to “for cetaceans”

Line 62: perhaps change to ‘in comparisons involving..’?

R: I wanted to keep relationships to be consistent with a previous sentence, but revised to say “allometric relationships that include data for other taxonomic groups.”

Line 81: “opportunistic collection… HAS provided”.

R: Changed as suggested.

Line 88: is the transfer of milk from the mother’s mammary gland to the offspring 100% efficient? The statements at lines 216 and 259 seem in contradiction with this. Also, does the energy that the mother invests in producing milk convert exactly to the energy contained in the milk? In general, I find this interchangeable terminology potentially confusing (and inappropriate, if there are inefficiencies); I would choose one of the two terms based on what is actually captured in these analyses, and stick with it throughout.

R: This sentence has been removed and I have gone through and changed terminology throughout as appropriate. Since I highlight in the introduction that milk energy intake is the vast majority of costs incurred during lactation, I do still refer to lactation costs when referencing the results more broadly (e.g., milk energy intake predictions can help parameterize daily lactation costs in bioenergetic models).

Lines 116-119: do you expect this methodological variation to impact the results in any relevant way?

R: This is a good question. The choice of equation used to translate CO2 production to energy expenditure can definitely impact the magnitude of the returned value. Similarly, when milk intake is estimated using just a single isotope, there are assumptions that need to be made about metabolic water production. This is not the case when you use DLW because then you can also get an estimate of MWP for each individual animal. So on the one hand, the answer is yes, but in terms of whether it impacts the results, it is hard to definitively say. My feeling is that for the most part this would just create more noise in the data and should be less influential with large sample sizes. I think it is probably most likely to influence the y-intercept for a given species, particularly at small sample sizes or when a species measurements are just represented by a single study. While it could have contributed to temporal patterns, the fixing of k largely avoided this (and indeed was one factor that drove that decision). To address this comment, I added some text to just state that yes, this can influence calculated values, and also included mention of this again in the limitations section to highlight the most likely place this is likely to affect results.

Line 135-138: just a note to underline that, while I understand the rationale for this necessary choice, this is a major simplification. In some species, the variation in lactation duration is large and, in fact, lactation duration is plastically adjusted depending on external conditions and, probably, condition of the mother and the offspring. This would be fine if sample sizes were large, but because the numbers are small, the specific animals that were sampled could have a large influence on the results. This is highlighted in the Discussion, but there could be some further reflection on its potential influence on the conclusions.

R: In the limitations section in the discussion, I now explicitly mention the most likely places that this particular issue, and other uncertainties, are likely to impact conclusions. For uncertainty in lactation duration, I highlight that it is likely to impact the specific timing of changes in costs during lactation (e.g. where in the relative time into lactation that inflections occur) but unlikely to impact broader taxonomic conclusions (e.g. the overall shape of temporal changes and the magnitude of change in response variables across the lactation period).

Line 145: if these data were compiled, it might be worth including them in a separate supplementary table anyway (even if they are not used in the analysis); having a record of published estimates, with rough lactation stage, could be a useful resource for other studies.

R: Agreed, however, I did not compile them because the compilation of data used in the analysis itself was very time intensive. I have added a sentence here highlighting where to find much of these data though.

Line 149: if I understand correctly, the models described in the next paragraphs were repeated 50 times (using the 50 simulated datasets), right? It might be worth stating this explicitly, and describing how the results from the 50 models were then combined when presenting the results.

R: Additional information has been provided to clarify that separate models were run for each replicate and are presented in their entirety in supplemental tables but averaged when presented in figures.

Line 154: I would spell out the rationale for using offspring-0.83 as the unit.

R: I have added a sentence to this effect with the citation here. The original citation with the 0.83 is from Oftedal’s thesis, but there is a more updated analysis by Riek that found 0.82 was the best value and so I just updated all of the analyses with that value since it is published.

Line 159: “data… WERE”.

R: Changed

Line 161: I would clarify that the random effect is on the intercept, and what that means in practice. On a related note: how reasonable is it to assume a single spline across all species in these diverse families (particularly for the order artiodactyls)? Could this contribute to the poor predictive power of the estimated relationships for cetaceans? I understand the rationale of this decision (line 166), and the caveat is highlighted in the Discussion, but this may require further reflection. The results (lines 286-287) also suggest that this assumption is likely too simplistic.

R: I added clarification to the methods about what this means, that it allows for species-specific intercepts but not smooths. While it would be ideal to allow for species-specific smooths, in practice this wasn’t possible, but I do not think this is what is contributing to the poor predictive power for cetaceans. As mentioned in a previous comment, I did add some additions to the discussion regarding some of the limitations and now explicitly add that this is unlikely to be the reason why predictions could not reproduce the observed cetacean pattern.

Line 162: Fixing k to avoid overfitting is controversial. While commonly used, it is not best practice. A better way to impose smoother relationships may be to increase the penalty for wiggliness using the gamma argument. I suspect it’ll make little difference in this case, though.

R: I did re-run all the models using gamma = 1.4 and removing the fixed k, and also with default settings (no fixed k and no changes in gamma). In most cases, this did not change the shape of the smooth. There were a few instances where additional wiggliness was introduced into the smooth when k was not fixed, which appeared to largely be a product of unequal distribution of species across the lactation interval and so was doubtful they were real. Because of this I chose to keep k fixed at 5 but added some additional description to better describe why. In response to another comment, I also added supplemental figures that show the actual data on top of the predicted smooth, which I think provides more transparency as to how predictions may or may not fit an individual species.

Line 178: Referring to these estimates as ‘data’ and ‘empirical estimates’ doesn’t sit well with me, for some of those cases. Some estimates are non-validated calculations largely based on strong assumptions around the value of many bioenergetic/morphometric parameters (e.g. for sea otters and the two cetaceans). It’s important to be clear that these are not direct measurements of milk intake or energy density. This caveat is spelled out below (199-202); still, I would avoid referring to these as ‘data’ or ‘empirical estimates’, and instead use ‘independent estimates’ throughout.

R: I changed throughout so these are all referred to as independent estimates. I also modified an existing sentence in the methods to clarify that independent estimates are based on some underlying empirical data, but are often relying on various assumptions to generate the estimate that was used for comparison. I think this clarification helps drive home the point that the reviewer is making here.

Line 270: Ditto. “The independent dataset was a bioenergetic model” is an oxymoron to me.ß

R: Addressed in above comment. This sentence now reads “Independent estimates were based on a bioenergetic model….”

Line 298, Table 1: I would report the units in the column names.

R: I added days to the lactation column header and a percentage sign to the coverage column headers. I did not add a unit to “n” because of space but I do think this one is more intuitive to readers. The definition for this is also found in the table header.

Lines 306-311 and 316-320: these are notes for the Tables, correct? It seems like an unusual way to describe Table content.

R: This is meant to be the table legend, which the journal species should occur below the table. I interpreted this as what you would find after the first sentence of the title but I agree that this looks weird. In looking at online versions, this appears normally so hopefully this should appear as expected in the actual published version.

Line 322 and 335: could you clarify what you mean by ‘conditional predictions’?

R: This is just meant to imply that predictions are made across a pre-defined set of values instead of averaging across the entire dataset. That said, it’s probably just clearer to say that they were made by species so I changed the titles to “Species-specific..”

Line 335, Figure 2: I imagine that a plot reporting the underpinning data would be hard to read. However, I suggest including family-specific plots of the predictions and data points coloured by species in supplementary material, to help the reader assess the goodness-of-fit of those models visually.

R: I added Supplemental figures where predictions for each species are plotted alongside the data that went into the model. It was too busy to show all species in a given family in one plot so I separated each species into their own subplot.

Line 492: “aRe likely”.

R: This has been fixed

Reviewer #2: This study represents a tremendous undertaking to leverage the use of existing data from marine and terrestrial mammals explore changes in milk intake rates and energy density throughout lactation, with a goal of developing predictive models that can be applied to bioenergetic models for data poor marine mammal species. There is a great need to explore that application, and this study handles complex and somewhat messy data in a comprehensive and organized way. The motivation and background for the study are clearly described, as are the methods including steps taken to convert data into consistent and comparable measures. Generalized GAMs are appropriate for handling these data. The use of independent data sources to explore model performance is a clever use of combining other data sources. The results are similarly clearly described. The discussion presented key findings that were supported by the analysis, and just as importantly, caveats and cautions of this analytical approach. While not the primary objective of this study, some taxonomic and life history comparisons were appropriately made to interpret findings. This was as rigorous

Attachment Submitted filename: Response to Reviewers.docx

# Editor report: Decision Letter 1

**DOI:** 10.1371/journal.pone.0352443.r003

**Authors:** Nathan Wolf, Nathan Wolf

11 Jun 2026

Predicting daily lactation costs of marine mammals for use in bioenergetic models

PONE-D-25-43306R1

Dear Dr. McHuron,

We’re pleased to inform you that your manuscript has been judged scientifically suitable for publication and will be formally accepted for publication once it meets all outstanding technical requirements.

Within one week, you’ll receive an e-mail detailing the required amendments. When these have been addressed, you’ll receive a formal acceptance letter and your manuscript will be scheduled for publication.

An invoice will be generated when your article is formally accepted. Please note, if your institution has a publishing partnership with PLOS and your article meets the relevant criteria, all or part of your publication costs will be covered. Please make sure your user information is up-to-date by logging into Editorial Manager at Editorial Manager® and clicking the ‘Update My Information' link at the top of the page. For questions related to billing, please contact billing support.

If your institution or institutions have a press office, please notify them about your upcoming paper to help maximize its impact. If they’ll be preparing press materials, please inform our press team as soon as possible -- no later than 48 hours after receiving the formal acceptance. Your manuscript will remain under strict press embargo until 2 pm Eastern Time on the date of publication. For more information, please contact onepress@plos.org.

Kind regards,

Nathan Wolf

Academic Editor

PLOS One

Additional Editor Comments (optional):

Dear Dr. McHuron,

Thank you for your patience during the long review process and for your thorough job in addressing the Reviewers' comments. The resulting manuscript will make a nice addition to PLOS One.

Reviewers' comments:

# Editor report: Acceptance letter

**DOI:** 10.1371/journal.pone.0352443.r004

**Authors:** Nathan Wolf, Nathan Wolf

PONE-D-25-43306R1

PLOS One

Dear Dr. McHuron,

I'm pleased to inform you that your manuscript has been deemed suitable for publication in PLOS One. Congratulations! Your manuscript is now being handed over to our production team.

At this stage, our production department will prepare your paper for publication. This includes ensuring the following:

* All references, tables, and figures are properly cited

* All relevant supporting information is included in the manuscript submission,

* There are no issues that prevent the paper from being properly typeset

You will receive further instructions from the production team, including instructions on how to review your proof when it is ready. Please keep in mind that we are working through a large volume of accepted articles, so please give us a few days to review your paper and let you know the next and final steps.

Lastly, if your institution or institutions have a press office, please let them know about your upcoming paper now to help maximize its impact. If they'll be preparing press materials, please inform our press team within the next 48 hours. Your manuscript will remain under strict press embargo until 2 pm Eastern Time on the date of publication. For more information, please contact onepress@plos.org.

You will receive an invoice from PLOS for your publication fee after your manuscript has reached the completed accept phase. If you receive an email requesting payment before acceptance or for any other service, this may be a phishing scheme. Learn how to identify phishing emails and protect your accounts at https://explore.plos.org/phishing.

If we can help with anything else, please email us at customercare@plos.org.

Thank you for submitting your work to PLOS ONE and supporting open access.

Kind regards,

PLOS ONE Editorial Office Staff

on behalf of

Dr. Nathan Wolf

Academic Editor

PLOS One
